# Methylome analysis of FTLD patients with TDP-43 pathology identifies epigenetic signatures specific to pathological subtypes

**DOI:** 10.1186/s13024-025-00869-2

**Published:** 2025-07-06

**Authors:** Cristina T. Vicente, Tejasvi Niranjan, Elise Coopman, Júlia Faura, Sara Alidadiani, Claudia Schrauwen, Billie J. Matchett, Bavo Heeman, Marleen Van den Broeck, Wouter De Coster, Thuy Nguyen, Julie S. Lau, Saurabh Baheti, Tim de Pooter, Peter De Rijk, Mojca Strazisar, Matt Baker, Mariely DeJesus-Hernandez, NiCole A. Finch, Cyril Pottier, Marka van Blitterswijk, Yan Asmann, Melissa E. Murray, Leonard Petrucelli, Andrew King, Claire Troakes, Safa Al-Sarraj, Robert A. Rissman, Annie Hiniker, Margaret Flanagan, Bret M. Evers, Charles L. White, Carlos Cruchaga, Rudolph Castellani, Jeroen G.J. van Rooij, Merel O. Mol, Harro Seelaar, John C. van Swieten, Björn Oskarsson, Robert Ross Reichard, Aivi T. Nguyen, Keith A. Josephs, Ronald C. Petersen, Nilüfer Ertekin-Taner, Bradley F. Boeve, Neill R. Graff-Radford, Sarah Weckhuysen, Dennis W. Dickson, Rosa Rademakers

**Affiliations:** 1 Department of Biomedical Sciences, University of Antwerp, Antwerp, Belgium; 2 VIB Center for Molecular Neurology, VIB, Antwerp, Belgium; 3 Division of Neuroscience, University of Manchester, Manchester, UK; 4 Department of Neuroscience, Mayo Clinic, Jacksonville, FL USA; 5 Genome Analysis Core, Mayo Clinic, Rochester, MN USA; 6 Department of Quantitative Health Sciences, Mayo Clinic, Rochester, MN USA; 7 Neuromics Support Facility, VIB Center for Molecular Neurology, VIB, Antwerp, Belgium; 8 Neuromics Support Facility, Department of Biomedical Sciences, University of Antwerp, Antwerp, Belgium; 9 Department of Neurology, Vanderbilt University Medical Center, Neuromuscular Division, Nashville, TN USA; 10 Department of Neurology, Washington University School of Medicine, St Louis, MO USA; 11 NeuroGenomics and Informatics Center, Washington University School of Medicine, St Louis, MO USA; 12 Department of Health Sciences Research, Mayo Clinic, Jacksonville, FL USA; 13 Department of Basic and Clinical Neuroscience, London Neurodegenerative Diseases Brain Bank, Institute of Psychiatry, Psychology and Neuroscience, King’s College London, London, UK; 14 Department of Clinical Neuropathology, King’s College Hospital NHS Foundation Trust, London, UK; 15 King’s College Hospital NHS Foundation Trust, London, UK; 16 Department of Neurosciences, University of California San Diego, San Diego, CA USA; 17 Veterans Affairs San Diego Healthcare System, San Diego, CA USA; 18 Department of Pathology, University of Southern California, Los Angeles, CA USA; 19 University of Texas Health Science Center San Antonio, San Antonio, TX USA; 20 Division of Neuropathology, University of Texas Southwestern Medical Center, Dallas, TX USA; 21 Department of Psychiatry, Knight Alzheimer Disease Research Center, Washington University School of Medicine, Saint Louis, MO USA; 22 Department of Pathology, Feinberg School of Medicine, Northwestern University, Chicago, IL USA; 23 Department of Neurology, Erasmus Medical Center, Rotterdam, The Netherlands; 24 Department of Clinical Genetics, Erasmus Medical Center, Rotterdam, The Netherlands; 25 Department of Neurology, Mayo Clinic, Jacksonville, FL USA; 26 Department of Laboratory Medicine and Pathology, Mayo Clinic, Rochester, MN USA; 27 Department of Neurology, Mayo Clinic, Rochester, MN USA; 28 Department of Neurology, Antwerp University Hospital, Antwerp, Belgium; 29 Translational Neurosciences, Faculty of Medicine and Health Science, University of Antwerp, Antwerp, Belgium

**Keywords:** Frontotemporal lobar degeneration, FTLD, TDP-43, FTLD-TDP, Pathological subtypes, Neurodegeneration, Epigenetics, DNA methylation, Methylome, Brain

## Abstract

**Background:**

In the last decade, the importance of DNA methylation in the functioning of the central nervous system has been highlighted through associations between methylation changes and differential expression of key genes involved in aging and neurodegenerative diseases. In frontotemporal lobar degeneration (FTLD), aberrant methylation has been reported in causal disease genes including *GRN* and *C9orf72*; however, the genome-wide contribution of epigenetic changes to the development of FTLD remains largely unexplored.

**Methods:**

We performed reduced representation bisulfite sequencing of matched pairs of post-mortem tissue from frontal cortex (FCX) and cerebellum (CER) from pathologically confirmed FTLD patients with TDP-43 pathology (FTLD-TDP) further divided into five subtypes and including both sporadic and genetic forms (*N* = 25 pairs per group), and neuropathologically normal controls (*N* = 42 pairs). Case-control differential methylation analyses were performed, both at the individual CpG level, and in regions of grouped CpGs (differentially methylated regions; DMRs), either including all genomic locations or only gene promoters. Gene Ontology (GO) analyses were then performed using all differentially methylated genes in each group of sporadic patients. Finally, additional datasets were queried to prioritize candidate genes for follow-up.

**Results:**

Using the largest FTLD-TDP DNA methylation dataset generated to date, we identified thousands of differentially methylated CpGs (FCX = 6,520; CER = 7,134) and several hundred DMRs in FTLD-TDP brains (FCX = 134; CER = 219). Of these, less than 10% are shared between pathological subgroups. Combining additional datasets, we identified, validated and replicated hypomethylation of *CAMTA1* in TDP-A potentially also impacting additional genes in the locus. GO analysis further implicated DNA methylation in myelination and developmental processes, as well as important disease-relevant mechanisms with subtype specificity such as protein phosphorylation and DNA damage repair in TDP-A, cholesterol biosynthesis in TDP-B, and protein localization in TDP-C.

**Conclusions:**

We identify methylation changes in all FTLD-TDP patient groups and show that most changes are unique to a specific pathological FTLD-TDP subtype, suggesting that these subtypes not only have distinct transcriptomic and genetic signatures, but are also epigenetically distinct. Our study constitutes an invaluable resource to the community and highlights the need for further studies to profile additional epigenetic layers within each FTLD-TDP pathological subtype.

**Supplementary Information:**

The online version contains supplementary material available at 10.1186/s13024-025-00869-2.

## Background

Frontotemporal Lobar Degeneration (FTLD) is the second most common type of young onset dementia (< 65 years) and comprises a genetically, clinically and pathologically heterogeneous group of neurodegenerative diseases that affect the frontal and temporal brain regions, leading to the impairment of basic human functions such as speech, behaviour and personality [1, 2]. The atrophy in these brain regions correlates with the accumulation of pathological lesions composed of inclusions with various compositions of disease proteins, of which TAR DNA-binding protein 43 (TDP-43) is the most common (FTLD-TDP) [3–5]. Based on the morphology and anatomical distribution of TDP-43-immunoreactive pathological neuronal cytoplasmic inclusions (NCIs) and dystrophic neurites (DNs), three main disease subtypes can be defined: FTLD-TDP types A-C. Briefly, in Type A patients, moderate to numerous NCIs and short DN are present predominantly in the upper cortical layers II/III; in Type B patients, moderate to numerous NCIs and sparse DNs are detected across all cortical layers; and in Type C patients, long dystrophic neurites are prevalent mainly in the upper cortices, whereas NCIs are not frequently found [6]. Apart from distinct histological patterns, FTLD-TDP subtypes are associated with distinct genetic causes and phenotypes such as disease presentation and duration. For instance, mutations in *GRN* invariably lead to FTLD-TDP type A while *C9orf72* repeat expansions are mainly linked to FTLD-TDP type B, which is often accompanied by amyotrophic lateral sclerosis (ALS). FTLD-TDP type C patients are usually sporadic with no known disease-causing genetic mutations. Furthermore, disease onset age is usually later in FTLD-TDP type A patients as compared to FTLD-TDP types B and C; FTLD-TDP type B patients die significantly younger; and FTLD-TDP type C patients have a longer disease duration [1, 7, 8]. These striking differences not only highlight the heterogeneity of disease phenotypes but also the multitude of pathomechanisms that may contribute to TDP-43 dysfunction and result in these different pathological FTLD-TDP subtypes.

With the discovery of the main causal genes and risk factors, including genetic factors specific to pathological FTLD-TDP subtypes, significant progress has been made to improve our understanding of the etiology of FTLD-TDP; however, the majority of patients remain genetically unexplained [9]. Transcriptomic studies conducted in FTLD-TDP and controls further revealed extensive transcriptional changes in FTLD-TDP brains [10, 11]; yet, the underlying molecular mechanisms driving FTLD pathology are not fully understood.

A possible contributing factor to the development of FTLD-TDP which has not been fully explored are epigenetic changes, i.e. modifications that modulate DNA function without altering the genetic sequence. Different types of epigenetic mechanisms exist, with the best studied being DNA methylation, which refers to the process by which a methyl group is added to a cytosine residue, transforming it into a 5-methylcytosine (5mC) [12]. This process mainly occurs on cytosines that precede a guanine nucleotide (CpG sites), which are scarce, spread across the genome and heavily methylated, except at CpG islands (CGI) which are clusters of high CpG density, usually between 0.5 and 2 kb long and mostly located at gene promoters where they regulate gene expression [12]. Interestingly, the brain is one of the tissues with highest levels of DNA methylation in the body [12]. In fact, in the last decades, several studies have highlighted the importance of epigenetics in the functioning of the central nervous system [13, 14], with an increasing body of work on brain DNA methylome showing an association between aberrant methylation patterns and differential expression of key genes involved in aging [15, 16] and neurodegenerative diseases such as Alzheimer’s disease (AD) [17–19], Parkinson’s disease (PD) [20–22] and Huntington’s diseases [23]. Specifically in FTLD, aberrant DNA methylation patterns were reported in causal genes such as *C9orf72* for which several studies have shown hypermethylation of a CGI located in the promoter region, as well as at the repeat expansion itself, in blood and brain tissue from FTLD and FTLD patients with concomitant ALS (FTLD-ALS) [24–26], and also in *GRN*, where promoter hypermethylation was reported in peripheral blood mononuclear cells (PBMCs), lymphoblasts and brain tissue from FTLD patients, and shown to negatively correlate with *GRN* expression [27, 28].

The aforementioned studies highlight the potential of investigating global epigenetic changes, and specifically DNA methylation changes, as potential risk factors and/or biomarkers for FTLD which remains largely unexplored, with current efforts limited to a single study [29].

To further investigate the genome-wide contribution of DNA methylation changes to FTLD-TDP pathology, we used reduced representation bisulfite sequencing (RRBS) [30] to profile the DNA methylation landscape in brain from neurologically normal subjects as well as several pathological groups of FTLD-TDP patients, either sporadic and presenting with the most common pathological disease subtypes A, B and C, or carrying a causal mutation in *GRN* or *C9orf72*. Furthermore, in addition to profiling these epigenetic changes in frontal cortex tissue, which is one of the most disease-relevant and affected brain regions in FTLD, we also studied cerebellum tissue from the same individuals, a less affected brain region that may be representative of early disease processes. Differential methylation analysis, followed by correlation with transcriptomic changes and enrichment analyses, identified several FTLD-TDP risk loci and dysregulated disease processes. Importantly, although we observe some commonalities, most of our findings are not only specific to pathological subgroups but are also brain region specific, highlighting a role for epigenetic modifications in the development of FTLD-TDP pathophysiology and its disease subtypes.

## Methods

### Sample selection

Human post-mortem samples from patients pathologically diagnosed with FTLD-TDP and neuropathologically normal controls were selected from the Mayo Clinic Brain Bank. The diagnosis of FTLD-TDP was made by an expert neuropathologist at one of the collaborating sites, based on the presence of TDP-43 positive inclusions as well as neuronal loss and gliosis in the frontal and temporal cortices. TDP-43 subtype was subsequently assigned for each patient based on the distribution of the neuronal cytoplasmic TDP-43-positive inclusions and dystrophic neurites in the cortical layers. A total of 167 frozen tissue pairs of frontal cortex (FCX) and cerebellum (CER) tissues were obtained together with clinical records on each subject, where available. Study subjects comprised two main groups: ***(i)*** FTLD-TDP patients subdivided into five pathological subgroups (*N* = 25 per group) including sporadic non-mutation carriers presenting with FTLD-TDP types A, B and C, *GRN* mutation carriers, and *C9orf72* repeat expansion carriers; and ***(ii)*** neuropathologically normal controls (*N* = 42) (Table 1).

**Table 1:** Study demographics

Pathological group	Group alias	Median age at death (IQR^a^) in years	% Female (*N*)	FCX-CER pairs^b^
FTLD-TDP Type A	TDP-A	83 (79.0–87.0)	52.0% (13)	25
FTLD-TDP Type B	TDP-B	68 (60.5–72.0)	48.0% (12)	25
FTLD-TDP Type C	TDP-C	74 (68.0-77.5)	40.0% (10)	25
FTLD-TDP *GRN* mutation carriers	TDP-GRN	68 (64.0–76.0)	56.0% (14)	25
FTLD-TDP *C9orf72* repeat expansion carriers	TDP-C9	67 (61.0-80.5)	44.0% (11)	25
Controls	CTRL	86.5 (79.8–89.3)	64.3% (27)	42

^a^IQR = Interquartile range

^b^FCX = Frontal cortex; CER = cerebellum

### Sample processing

Manual DNA extraction from frozen tissue (50-75 mg from FCX and 30-40 mg from CER) was performed using the Autogen 245T Reagent Kit (AutoGen, Holliston, MA) following manufacturers’ instructions. Extracted DNA was quantified using both a Qubit 2.0 Fluorometer (ThermoFisher Scientific, Waltham, MA) and the Quant-iT Picogreen dsDNA assay kit (Invitrogen, Carlsbad, CA). Samples were randomized and 500ng of DNA from each sample was transferred into 96-well plates and shipped to the Mayo Clinic Medical Genome Facility in Rochester (Minnesota, USA) for processing and sequencing.

### RRBS

RRBS libraries were prepared from 125ng of genomic DNA using the Ovation RRBS Methyl-Seq System (Tecan Genomics, Redwood City, CA). Briefly, dsDNA was digested with Msp1 and indexed methylated adaptors were ligated to the digested fragments with T4 DNA ligase. Ligated DNA was repaired with Final Repair mix and bisulfite conversion was performed using the EZ-DNA Methylation Kit (Zymo Research, Irvine, CA). Bisulfite modified products were then amplified with PCR and purified with AMPure beads. The concentration and size distribution of the completed libraries were determined using Fragment Analyzer (Agilent, Santa Clara, CA) and Qubit fluorometry (ThermoFisher Scientific, Waltham, MA). Completed libraries were multiplexed at eight samples per lane and 20% commercially prepared PhiX library (Illumina, San Diego, CA) was added to increase base diversity and improve sequencing quality. Samples were sequenced following Illumina’s standard protocol using the Illumina cBot and HiSeq 3000/4000 PE Cluster Kit. A custom Read 1 primer, MetSeq Read 1 was spiked in with the Illumina Read 1 primer. The flow cells were sequenced as 51 × 2 paired end reads on an Illumina HiSeq 4000 using the HiSeq 3000/4000 sequencing kit and HCS v3.4.0.38 collection software. Base-calling was performed with Illumina’s RTA version 2.7.7.

### RRBS alignment and quality control (QC)

FASTQ files were trimmed to remove adaptor sequences and reads with less than 15 bp were discarded. Trimmed FASTQs were then aligned to the human genome reference GRCh38/hg38 with bwa-meth using default parameters [31]. Extraction of methylation information was done using MethylDackel [github: dpryan79/MethylDackel] with discordant reads retained, minimum mapping quality (-q) set to 35, and minimum depth of coverage (-d) set to 5. Alignments were custom genotyped with samtools mpileup using reads with mapping quality ≥ 30 and base calls with quality ≥ 30 in non-repetitive regions (region inversion of hg38 repeat mask, UCSC Genome Browser). Common variants (MAF ≥ 1% in gnomAD) that are not subject to bisulfite conversion effects (A > T substitutions) were retained for genotype comparisons between samples. Coverage maps of the X and Y chromosomes were used to approximate sex and compare to the ascertained sex (Supp. Figure 1A). Methylation values were compared at all positions with at least 5x coverage in all samples using principal components analysis. The first six principal components were assessed for inappropriate outliers and grouping (Supp. Figure 1B). After QC, removed samples from the study included three samples due to discordant matching between tissue pairs, six samples as ethnic outliers, three samples due to discordance with ascertained sex (one of which was also considered an ethnic outlier), one sample as a PCA outlier (also due to sex discrepancy and familial stratification), and six CER samples due to PCA grouping with FCX (Supp. Table 1). After QC, 309 samples were retained with *N* = 158 FCX and *N* = 151 CER samples.

### Differential methylation analyses

Four differential methylation analyses were conducted in parallel at the level of: (***i)*** individual CpG positions (CpGs); (***ii)*** gene promoters, defined as ± 500 bp of a transcriptional start site (TSS); (***iii)*** genome-wide, employing a 500 bp sliding window approach with 250 bp shifts generated by MethylKit [32]; and (**iv**) genome-wide, employing variable-length regions refined by the DMRfinder package [33], with parameters r = 5 and s = 20. Analysis of differential methylation was performed by fitting a generalized linear model and performing a likelihood-ratio test using the edgeR package [34] and using sex and age at death as covariates. Separately for each tissue (FCX and CER), 5 individual group comparisons were performed with each TDP subgroup (TDP-A, TDP-B, TDP-C, TDP-C9 and TDP-GRN) against controls, as well as two additional comparisons where TDP subgroups were combined (group ABC: including all genetically unexplained FTLD-TDP groups [TDP-A + TDP-B + TDP-C]; and group TDP: which included all FTLD-TDP groups irrespective of genetic etiology) and compared to controls. Each comparison was performed separately per tissue and at the level of individual CpGs, gene promoters, sliding window (methylKit) regions, and DMRfinder defined regions. Significant loci were annotated using the GENCODE v.44 basic gene annotation set, FANTOM5 enhancers, and CpG islands using the ‘annotatr’ R package [35, 36]. Individual significant CpGs were annotated for overlap with known common variants with a population frequency > 1% using dbSNP build 151 [37]. If a locus spanned multiple regions within a given gene, only one genomic context annotation was retained, with priority defined by the following hierarchy: promoter > untranslated regions (5’-UTR > 3’-UTR) > coding sequence (CDS) > exon > intron > intergenic. CDS, exon and intron terms were further merged and defined as gene body. If several transcripts from the same gene overlapped, only one annotation was retained, following the same hierarchy. If transcripts from different genes overlapped, only one annotation was retained if the genomic context was the same for both genes, otherwise we retained one annotation per gene in the locus.

### Gene ontology (GO) enrichment analyses

GO enrichment analysis of top hit genes was performed using the enrichR library R package [38]. Specific attention was paid to the Gene Ontology sets Biological Process and Molecular Function (2023 knowledge base) [39]. For each genetically unexplained pathological group (TDP-A, TDP-B, TDP-C and combined group ABC), we used the lists of gene symbols annotated to all differentially methylated CpGs, promoter regions and genome-wide differentially methylated regions (DMRs; defined by methylKit and DMRfinder) as input for Gene Ontology (GO) analyses. Top enrichment terms were restricted to Pvalue < 0.05. All other enrichR parameters were kept at default.

### GO clustering

GO terms are connected in a directed acyclic graph [39]. Gene overlap between biologically related and connected terms increases the likelihood that these connected terms may be co-enriched. While this is not statistically informative, it allows for clustering of co-enriched GO terms based on both their biologically relevant connections and change patterns across FTLD-TDP subtypes and brain regions. To accomplish this clustering, unique significant GO terms with two or more genes, derived from any of the pathological groups (TDP-A, TDP-B, TDP-C and combined group ABC) or tissues (FCX and CER) were collapsed together and then clustered into the largest set of terms that are unbroken within the GO graph. Performing this analysis generated clusters ranging in term sizes of 4 to 1,811. To improve the clarity of related terms, clusters larger or equal to 25 terms were split into smaller clusters by iteratively removing the highest parental term. Where possible, these higher parental terms were then re-merged with an adjacent cluster that is smaller than the 25-term limit. If more than one adjacent cluster exists, priority is given to smaller clusters with higher semantic similarity. We visualized clusters with four or more GO terms to compare graph enrichments across FTLD-TDP subtypes and tissues. Clustering and visualization was performed using a custom R script, GOgraphClust, taking advantage of the R packages enrichR [38], GOfuncR [40], ggraph [41], and igraph [42].

### Targeted bisulfite sequencing for *GFPT2* validation

As the gold standard technique, targeted bisulfite sequencing was performed to confirm methylation levels in the 500 bp DMR within *GFPT2* (Glutamine-fructose-6-phosphate transaminase 2; chr5:180,313,751 − 180,314,251), in TDP-C and controls. From each group we selected four samples that we classified according to methylation levels measured by RRBS, as high (> 80%, N = 1 per group), intermediate (N = 2 per group) and low (< 20%; N = 1 per group) methylation across the *GFPT2* DMR, and for which we had DNA available from the RRBS cohort. From each sample, 500ng of DNA was bisulfite converted using the EZ DNA Methylation-Lightning kit (Zymo Research, Irvine, CA) following manufacturers’ instructions. 50ng of bisulfite converted DNA from each sample were then used to amplify the *GFPT2* DMR by polymerase chain reaction (PCR), in reactions containing 10x buffer (Invitrogen, Carlsbad, CA), dNTPs (10mM, Invitrogen, Carlsbad, CA), MgCl2 (50mM, Invitrogen, Carlsbad, CA), bisulfite specific primers (10µM, Integrated DNA Technologies, Coralville, IA) and Platinum Taq DNA Polymerase (1µl, Invitrogen, Carlsbad, CA). PCR amplification was performed in a Veriti 96-well fast thermal cycler (Applied Biosystems, Waltham, MA) as follows: 5min at 94°C, 40 cycles of 30sec at 94°C, 30sec at 60°C, and finally 45sec at 72°C. Bisulfite specific primers were designed with Bisulfite Primer Seeker (5’-AATTTAAGAGGGGAGGGGAAA-3’; 5’-TAACACACCTTAAATATAAAAATCAACC-3’; Zymo research, Irvine, CA). PCR products were then loaded on a 1% agarose gel and bands excised based on the correct molecular weight. DNA was extracted from the excised gel bands using the NucleoSpin PCR Clean-Up and Gel extraction kit (Macherey-Nagel, Düren, Germany) according to the manufacturers’ protocol. The purity and concentration of the extracted DNA was determined using a NanoDrop 1000 spectrophotometer (ThermoFisher Scientific, Waltham, MA). DNA fragments were then ligated into the highly efficient pGEM-T Vector using the pGEM-T Vector Systems kit (Promega, Madison, WI) at a 3:1 molar ratio insert: vector. Ligation reactions were incubated overnight at 4 °C and transformed into competent JM109 bacteria using standard bacterial transformation protocols. Transformed bacteria were plated in Luria-Bertani (LB) agar plates containing ampicillin (Amp) as well as isopropylthio-β-galactoside (IPTG; 100mM; Life Technologies, Carlsbad, CA) and 5-bromo-4-chloro-3-indolyl-beta-D-galacto-pyranoside (X-Gal; 40 mg/mL; Life Technologies, Carlsbad, CA), to allow for white/blue colony screening, and incubated at 37 °C overnight. From each sample, *N* = 6 white colonies were picked and expanded in LB/Amp medium, in a shaking incubator at 37 °C overnight. The following day, bacteria were harvested, and plasmid DNA was purified from each culture using the NucleoSpin Plasmid EasyPure MiniPrep kit (Macherey-Nagel, Düren, Germany) following manufacturers’ instructions. The purity and concentration of the extracted DNA was determined using a NanoDrop spectrophotometer 1000 (Thermo Fisher, Waltham, MA). 200ng of plasmid DNA from each colony was then processed for sanger sequencing, in reactions containing BigDye terminators and universal M13 sequencing primers, in a PeqSTAR 96X universal thermal cycler (PeqLab Biotechnologies GmbH, Erlangen, Germany) as follows: 2 min at 96 °C, 25 cycles of 10 min at 96 °C, 5 min at 50 °C, and 4 min at 60 °C. Sanger sequencing was performed in-house on a 3730xl DNA Analyzer (Applied Biosystems, Waltham, MA) at the Neuromics Support Facility.

### Targeted bisulfite sequencing data analysis

CLC Main Workbench 22.0 (Qiagen, Hilden, Germany) was used to visualize sequencing chromatograms and trim sequences. Trimmed bisulfite converted sanger sequences were then analyzed using the DNA methylation analysis tool BiQ Analyzer [43]. QC of each bisulfite sequence was performed as part of the analysis and for each covered Cytosine (*N* = 45) methylation was accessed. Methylation percentages per sample were calculated as a ratio between the total number of methylated CpGs/total number of CpGs, for all positions and across all clones from a given sample (*N* = 6). Results are shown as a standard lollipop-style plot, where each row represents a clone and each dot represents a profiled Cytosine.

### Short-read RNA sequencing data analysis

Transcriptomic data was available in-house from bulk FCX and CER tissue [10] and used in this study either: (**i**) alone to show expression of selected genes in all FTLD-TDP patients as compared to control subjects; or (**ii**) in combination with methylation data for a subset of individuals for which both measures where available, to assess correlation between the two datasets for selected genes. In both instances, normalized counts were used as the quantification measure of gene expression. Briefly, RNA from FCX (*N* = 127 FTLD-TDP and *N* = 22 neuropathologically normal controls) and CER (*N* = 160 FTLD-TDP and *N* = 20 neuropathologically normal controls) was extracted using the RNeasy Plus mini kit (Qiagen, Hilden, Germany). RNA quality and quantity was assessed using an Agilent 2100 Bioanalyzer and the RNA Nano Chip (Agilent Technologies, Santa Clara, CA), and only samples with an RNA integrity number (RIN) above seven were included in the study. Library preparation was performed using the Illumina TruSeq mRNA v.2 prep and sequenced as 101 bp paired-end on a HiSeq4000 (Illumina, San Diego, CA). Raw RNA-sequencing reads were aligned to the human reference genome (GRCh38) using the spliced transcripts alignment to a reference (STAR, v.2.5.2b) [44]. Library quality assessment was performed using the RSeQC package (v.3.0.0) [45]. Gene-level expression was quantified using the featureCounts command in the Subread package (v.1.5.1) [46]. Variance-stabilizing transformation counts were obtained using the vst function of the DESeq2 R package [47]. Genes with fewer than 20 samples with at least 10 supporting reads were excluded. After QC, six FCX samples were removed (*N* = 3 FTLD-TDP and *N* = 3 neuropathologically normal controls), and a total of 23,458 genes in FCX and 23,887 genes in CER, were retained for analysis.

### Long-read DNA sequencing with Oxford Nanopore Technologies (ONT)

Long-read ONT data from FCX tissue from TDP-A and controls was used to perform differential methylation analyses for the *CAMTA1* DMR (chr1:7574506–7574692). A total of 155 samples were sequenced, comprising: (**i**) a ‘validation cohort’ including 53 individuals overlapping with the RRBS cohort (*N* = 28 neurologically normal controls and *N* = 25 TDP-A); and (**ii**) a ‘replication cohort’ including 102 additional samples sourced from both the Mayo Clinic Brain Bank as well as additional independent collaborators from the international FTLD-TDP whole genome sequencing consortium with at least five samples of TDP-A and/or controls available. The replication cohort included 22 samples from neuropathologically normal controls (median age at death = 72 years (60.75–80.5); 54.5% females; *N* = 10 from the Erasmus Medical Center; *N* = 6 from Northwestern University; *N* = 3 from the Mayo Clinic Brain Bank; and *N* = 3 from the University of California San Diego) and 80 TDP-A cases (median age at death = 78.5 years (67.5–84.0); 35% females; *N* = 47 from the Mayo Clinic Brain Bank; *N* = 11 from the University of Texas Southwestern Medical Center; *N* = 8 from Washington University; *N* = 5 from King’s College London; *N* = 4 from the Erasmus Medical Center; *N* = 2 from the University of California San Diego; and *N* = 3 from Northwestern University). For each subject, DNA was extracted from frozen FCX tissue with the Nanobind PanDNA kit (hmwDNA) (PacBio, California, USA) following manufacturer’s recommendations. Library preparation was performed using ONT SQK-LSK 109 and SQK-LSK 110 kits (Oxford Nanopore Technologies, Oxford, UK), followed by sequencing using a PromethION24 sequencer and R9.4.1 flow cells (Oxford Nanopore Technologies, Oxford, UK). After sequencing, base calling was performed using guppy v6.3.7 with the dna_r9.4.1_450bps_modbases_5hmc_5mc_cg_hac_prom model (Oxford Nanopore Technologies, Oxford, UK). Alignment to the human reference genome (GRCh38) was performed with minimap2 v2.24 [48], and haplotype phasing was done using longshot v0.4.5 [49]. Modification data for CpG sites in the *CAMTA1* DMR was extracted per haplotype from the combined strands using modkit version v0.2.4 (Oxford Nanopore Technologies, Oxford, UK). Modkit output files were further processed, filtering for methylation calls and sites with minimum coverage of 5 reads. Differential methylation analysis for the *CAMTA1* DMR between TDP-A and neuropathologically normal controls, was performed as for the RRBS data, using the edgeR package [34] and including sex and age at death as covariates.

### Generation and long-read cDNA sequencing with ONT of *TARDBP* knockdown human pluripotent stem cell (iPSC)-derived neurons

Data was generated and processed as previously described [50]. Briefly, a human iPSC line from a neurologically normal donor (WTSIi040-A) was sourced from the European Bank for induced pluripotent Stem Cells (EBiSC) and differentiated into cortical glutamatergic projection neurons using an established protocol [51] with minimal adaptations. For final plating as cortical neurons, cells were dissociated and seeded at a density of 750,000 cells/well. At DIV126, neurons were transduced independently for 48 h with three different lentiviral particles packaged with a short hairpin RNA (LV-shRNA) to either knock-down (KD) *TARDBP* (*shTARDBP*) (targeting different regions of the gene leading to increasing levels of silencing) or a scramble control. Five days post-transduction, cells were dissociated, pelleted and frozen until further processing. RNA was extracted from the frozen pellets using the RNeasy Mini kit (Qiagen, Hilden, Germany), followed by qPCR to assess *TARDBP* expression and KD efficiency. Total RNAs extracted from iPSC-derived neurons were then subjected to a polyA enrichment step, using the Poly(A) RNA Selection Kit (V1.5, Lexogen, Vienna, Austria). cDNA libraries were prepared using the direct cDNA sequencing Kit with native barcoding (Oxford Nanopore Technologies, Oxford, UK) and sequenced on a PromethION24 sequencer (Oxford Nanopore Technologies, Oxford, UK). Base calling was performed using guppy v3.4.5, alignment to the hg38 human reference genome was performed with minimap2 [52], and transcript identification and quantification was performed with IsoQuant [53]. Novel genes were excluded, and expression values at transcript level were normalized using DESeq2 [47].

### Statistical analyses and figures

Normality of methylation and transcriptomic datasets for the specified genes was assessed by using the Shapiro-Wilk test or the Kolmogorov-Smirnov test, depending on sample size, and visual inspection of frequency distribution histograms. When a normal distribution was observed, testing between two groups was performed using an unpaired t-test, and between multiple groups using an ordinary one-way ANOVA. In the cases where distribution was not normal, a Mann-Whitney test was used to compare two groups, and a Kruskal-Wallis test for multiple comparisons. For post-hoc comparisons, Bonferroni correction was used to adjust for multiple testing. To test correlations between methylation and gene expression values, a Pearson correlation test was used. All statistical analyses were performed using RStudio v4.3.3 or GraphPad Prism 10.0.3 (GraphPad Software, Boston, MA) with a significance threshold of 0.05. Figures were created using the R package ggplot2 [54], Biorender (Toronto, Canada) and Adobe Illustrator 2024 (Adobe Systems, San Jose, CA).

## Results

### Thousands of differentially methylated CpGs characterize individual FTLD-TDP pathological subtypes

**Fig. 1:**
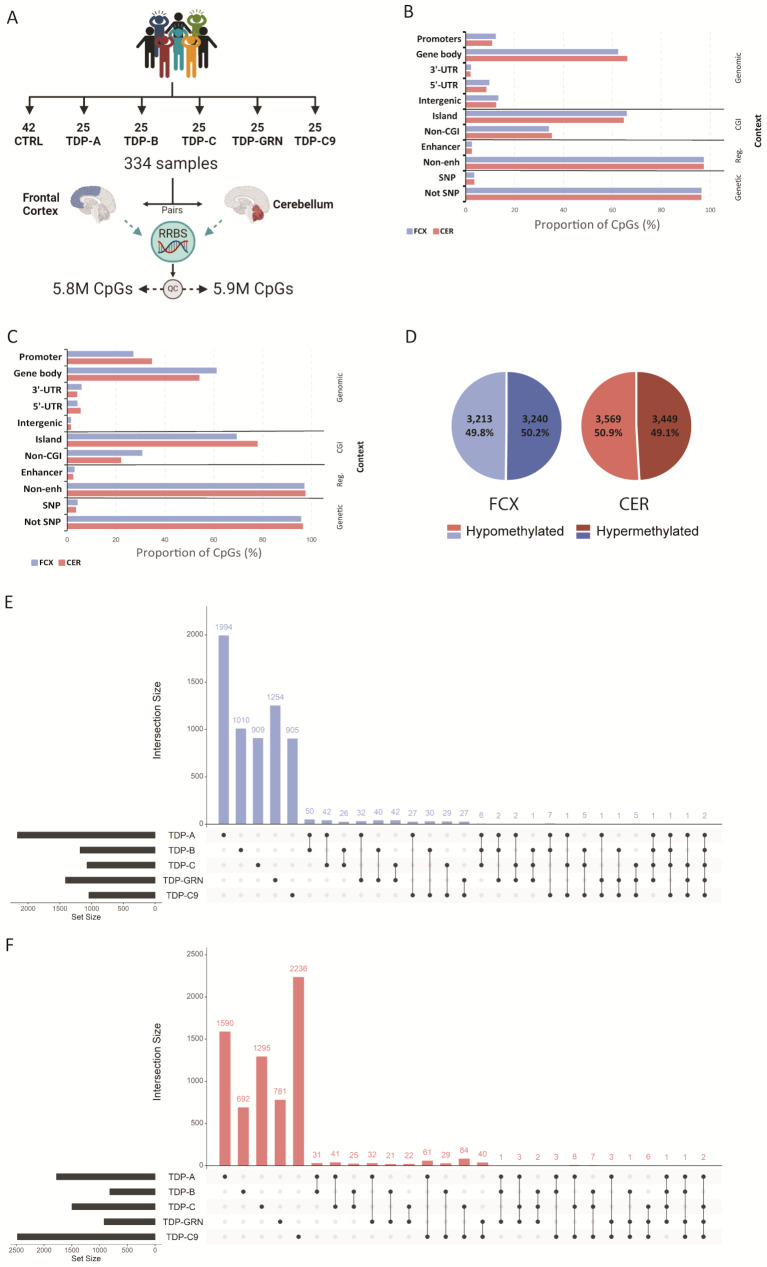
RRBS identifies thousands of differentially methylated CpGs in brain tissue from FLTD-TDP patients. Study outline (**A**). Proportion of CpGs in different contexts including: genomic region, which relates to the CpG position relative to the annotated genes; overlap with a known CpG island (CGI); overlap with regulatory features (enhancers, enh); and genetic context considering only common single nucleotide polymorphisms (SNP). Graphs show the proportion of CpGs in both FCX (blue bars) and CER (red bars) including either all CpGs retained in the study (**B**) or only significantly differentially methylated sites across all patient groups (**C**). Distribution of differentially hypomethylated (light shades) and hypermethylated (dark shades) CpGs across all groups, in FCX (left; blue graph) and CER (right; red graph) (**D**). Upset plot showing the number of unique and overlapping CpGs in each pathological group, considering all differentially methylated CpGs in FCX (**E**) and CER (**F**)

RRBS was performed to generate DNA methylation profiles from pairs of frozen post-mortem FCX and CER from FTLD-TDP patients (FTLD-TDP types A, B and C, *GRN* mutation carriers and *C9orf72* repeat expansion carriers) and neuropathologically normal controls (Fig. 1A). After QC, 5,819,868 CpGs in FCX and 5,936,364 in CER were included in the analyses. 90% of the total number of retained CpGs overlapped between both tissues, with similar distributions with respects to genomic region, CpG island and regulatory element context (Fig. 1B). Differential methylation analysis was then performed at the CpG site level in both tissues, between each individual pathological subgroup and controls (Supp. Tables 2 and 3). Across all groups, we found 6,453 differentially methylated CpG sites (FDR < 0.05) in FCX and 7,018 in CER. In both brain regions, the majority of differentially methylated CpGs were in a gene body (61.1% in FCX and 54.1% in CER), followed by gene promoters (27.1% in FCX and 34.7% in CER), 3’-UTRs (5.9% in FCX and 4.1% in CER), 5’-UTRs (4.2% in FCX and 5.5% in CER), and a small proportion of intergenic CpGs (1.6% in both FCX and CER; Fig. 1C). In each tissue we found approximately the same number of CpGs to be hypo- and hypermethylated in FTLD-TDP patients, when compared to controls (Fig. 1D). Interestingly, the vast majority of differentially methylated CpGs we identified were unique to a disease subtype, with less than 10% of sites shared between two or more individual patient subgroups in both FCX (381 CpGs representing 6%; Fig. 1E) and CER (424 sites representing 6%; Fig. 1F). Of the overlapping CpGs in FCX, only six were found to be differentially methylated only in genetically unexplained groups of patients (TDP-A, TDP-B and TDP-C), annotated to *CDH15*, *FN3KRP*, *HS1BP3*, *CYP2W1*, *NDUFAF6*, *TP53INP1* and *ZIC3*, whereas only two CpGs (within *PLCB3* and *UBE2A)* were found differentially methylated across all pathological subtypes. In CER, no CpG sites were found in common between only genetically unexplained subgroups or all patients. Although we found that CpG positions were not commonly shared between disease groups, we did identify overlaps when analyzing the intersection of annotated genes from all differentially methylated CpGs. We found that 28.2% of genes overlapped between the different groups in FCX (1,327 genes; Supp. Figure 2 A) and 29.4% in CER (1,592 genes; Supp. Figure 2B). In FCX, the largest overlap was observed between TDP-A and all other disease subtypes, the majority being shared with TDP-GRN and TDP-B. Furthermore, we identified 25 genes in FCX and 20 in CER harboring differentially methylated CpG sites only within the sporadic patient groups (none of which was in common between both tissues), and 41 genes in FCX and 16 in CER where differentially methylated CpG sites were found across all patient groups, of which four were detected in both brain regions (*HDAC4*, *PRDM16*, *PTPRN2* and *RASA3*, Supp. Tables 2 and 3). When analyzing the genes containing the most differentially methylated CpGs (≥ 5 CpGs) within each pathological subgroup, we found that in FCX, the TDP-A group had the highest number of such genes (*N* = 16), followed by TDP-GRN (*N* = 5), TDP-C (*N* = 5), TDP-B (*N* = 2) and finally TDP-C9 (*N* = 1) (Supp. Table 2). In CER however, we found the TDP-C9 group to have the highest number of such genes (*N* = 12), followed by TDP-A (*N* = 8), TDP-C (*N* = 7), and lastly TDP-GRN (*N* = 1) with none in TDP-B (Supp. Table 3). We next sought to investigate shared epigenetic mechanisms between patients, by combining groups of patients and comparing those to controls (genetically unexplained group ‘ABC’ including TDP-A/B/C and group ‘TDP’ including all TDP patients). We found that group ‘ABC’ only contributed 54 unique CpG sites in FCX and 108 in CER, representing 24 and 58 unique genes in FCX and CER, respectively (Supp. Tables 2 and 3). Group ‘TDP’ further contributed only a few additional unique CpGs with 13 in FCX and 8 in CER, representing 10 unique genes in FCX and 5 in CER, further supporting the specificity of findings to pathological subtypes, rather than shared disease mechanisms (Supp. Tables 2 and 3). Finally, to determine whether our findings are also brain region specific, we compared FCX to CER and found that only 64 CpG sites are common between brain regions across all disease groups (Supp. Tables 2 and 3). In terms of genes harboring differentially methylated CpGs, we also found a limited overlap between tissues, with 406 genes in TDP-A, 141 in TDP-B, 200 in TDP-C, 151 in TDP-GRN and 301 in TDP-C9, supporting the specificity of disease-associated methylation patterns not only to pathological subtypes but also to the brain region.

### RRBS identifies differentially methylated CpGs in known FTLD genes

Next, we employed a targeted approach to investigate the presence of differentially methylated CpGs (FDR < 0.05) in both FCX and CER within known FTLD genes [8], including *CHCHD10* [55], *CHMP2B* [56], *CSF1R* [57], C*9orf72* [58, 59], *FUS* [60], *GRN* [61, 62], *hnRNPA1* [63], *hnRNPA2B1* [63], *LRRK2* [64], *MAPT* [65], *OPTN* [66], *SQSTM1* [67], *TARDBP* [5], *TBK1* [66], *TIA1* [68], *UBQLN2* [69], *VCP* [70], as well as the recently implicated *UNC13A* [71–73], *TNIP1* [73] and *ANXA11* [74, 75]. We also included three additional genes previously reported to be differentially methylated in FTLD patients: *SERPINA1* specifically in the *C9orf72* repeat extension carrier group [76], and *NFATC1* and *OTUD4* which were reported across different FTLD pathological subtypes [29]. Overall, only few differentially methylated CpGs were found in these genes (Table 2); however, in the case of *GRN* and *C9orf72* the previously identified differentially methylated regions in these genes were poorly covered in our study. Furthermore, and despite none of them overlapping with the previously reported CpG in intron 9, we did find that *NFATC1* harbored numerous differentially methylated CpGs across multiple patient subgroups (Supp. Figure 3A). Of the differentially methylated CpGs in *NFATC1* that we identified in the FCX, several showed high regulatory potential due to their location within the gene (promoter and both 5’- and 3’-UTRs). Given the previously reported finding that the expression of *NFATC1* is increased in FCX from FTLD patients, we investigated *NFATC1* expression in our previously generated bulk RNA sequencing dataset [10] and also found higher expression of *NFATC1* in FCX from FTLD-TDP patients, when compared to controls (Supp. Figure 3B). We next tested the correlation between methylation levels at each differentially methylated CpG site in FCX and *NFATC1* expression, in all FTLD-TDP patients for which both datasets were available, and found that methylation levels at the 5’-UTR CpG negatively correlated with the expression level of *NFATC1* (*r*= -0.29; *P* = 0.0034; Supp. Figure 3C) suggesting that in addition to the previously reported intronic CpG, this 5’-UTR CpG may also play a role in regulating *NFATC1* in FCX.

**Table 2:** Distribution of significantly differentially methylated CpGs within known FTLD genes

Brain region^a^	Gene	TDP-A	TDP-B	TDP-C	TDP-GRN	TDP-C9
FCX	*VCP*	1	1	-	-	-
FCX	*LRRK2*	1	-	-	-	-
FCX	*ANXA11*	1	-	-	-	-
CER		1	-	-	-	-
FCX	*NFATC1*	1	1	-	1	1
CER		1	2	4	-	7
CER	*TNIP1*	-	-	1	-	1
CER	*OTUD4*	-	-	1	-	-
CER	*FUS*	-	-	-	-	1
CER	*hnRNPA2B1*	-	-	-	-	1

^a^FCX=frontal cortex and CER = cerebellum

### Promoter level differential methylation analysis identifies 12 promoter loci in FCX and 8 in CER

The single-base resolution of our data allows the investigation of individual CpG sites, much like array-based studies where methylation is profiled at single CpG sites and with only a few sites being profiled per gene; however, CpGs are most often clustered within CpG islands located in genomic areas with likely functional significance. As such, we sought to investigate whether aberrant methylation patterns are observed in CpG islands, in the brain of FTLD-TDP patients. For this, CpG sites were grouped into regions, and differential methylation analysis at the region level was performed. First, we included only loci located within gene promoters (defined by location ± 500 bp from the TSS) and performed differential methylation analysis in FCX and CER separately. We identified 12 differentially methylated regions (DMRs) in FCX and eight in CER, annotated to the promoters of 15 and 13 genes, respectively (Tables 3 and 4). In both tissues, we identified both hypo- and hypermethylated loci (67% hypo- and 33% hypermethylated in FCX; 50% hypo- and 50% hypermethylated in CER). None of the loci overlapped between brain regions and interestingly, promoter DMRs were mostly identified in genetically unexplained FTLD-TDP patients (subtypes TDP-A, TDP-B and TDP-C in FCX; subtype TDP-C in CER). Finally, in FCX only two loci were found in common between patient groups (*TRIM34* and *LINC01954*) whereas in CER no shared loci were identified.

**Table 3:** Results from the differential methylation promoter analysis in frontal cortex

Genomic position^a^	Annotated gene^b^	Group	LogFC^c^	FDR^d^
chr11:5,619,444–5,620,444	*TRIM34*	TDP-B	-3.5093	7.612E-05
chr11:5,619,444–5,620,444	*TRIM34*	TDP	-2.2328	0.00088
chr11:5,619,444–5,620,444	*TRIM34*	ABC	-2.2417	0.00095
chr22:44,172,456–44,173,456	*PARVG*	TDP-C	1.1360	0.00286
chr22:41,998,288–41,999,288	*WBP2NL*	TDP-A	-0.9169	0.00376
chr2:10,877,767–10,878,767	*LINC01954*	ABC	1.2295	0.0157
chr11:5,619,444–5,620,444	*TRIM34*	TDP-C	-2.6145	0.0158
chr13:63,843,625–63,844,625	*LOC102723968*	TDP-C	-2.2257	0.0158
chr2:61,017,755–61,018,755	*PUS10; PEX13*	TDP-B	-1.1393	0.0187
chr12:2,003,957–2,004,957	*DCP1B*	TDP-B	2.3815	0.0187
chr19:11,495,122–11,496,122	*MIR7974*	TDP-C9	-0.9181	0.0266
chr13:36,297,314–36,298,314	*CCDC169; CCDC169-SOHLH2*	TDP-C9	-1.1253	0.0266
chr5:140,840,687–140,841,687	*PCDHA8*	TDP-B	0.6537	0.0336
chr1:112,850,143–112,851,143	*LINC01356; LINC01357*	TDP	-1.2340	0.0379
chr3:48,196,064–48,197,064	*MIR4443*	TDP-A	-2.3393	0.0492
chr2:10,877,767–10,878,767	*LINC01954*	TDP-C	1.3629	0.0424

^a^GRCh38 assembly

^b^Nearest gene according to GENCODE v44

^c^FC = Fold change. Negative and positive values denote hypo- and hypermethylation, respectively, in patients compared to controls

^d^FDR = False discovery rate

**Table 4:** Results from the differential methylation promoter analysis in cerebellum

Genomic position^a^	Annotated gene^b^	Group	logFC^c^	FDR^d^
chr7: 133,252,567–133,253,567	*EXOC4*	TDP-C	-1.8203	0.0276
chr12: 56,157,859–56,158,859	*MYL6; MYL6B-AS1*	TDP-C	-0.7088	0.0276
chr14: 56,811,506–56,812,506	*OTX2-AS1*	TDP-C	0.7042	0.0276
chr17: 5,468,592–5,469,592	*DHX33; DHX33-DT*	TDP-C	-0.7380	0.0276
chr19: 35,741,952–35,742,952	*IGFLR1*	TDP-C9	3.1739	0.0386
chr19: 36,013,713–36,014,713	*ALKBH6; CLIP3; LOC101927572*	TDP-GRN	-2.2226	0.0085
chr19: 57,279,834–57,280,834	*ZNF460; ZNF460-AS1*	TDP	1.1382	0.0405
chrX: 45,730,588–45,731,588	*MFFP3*	TDP	1.0859	0.0481

^a^GRCh38 assembly

^b^Nearest gene according to GENCODE v44

^c^FC = Fold change. Negative and positive values denote hypo- and hypermethylation, respectively, in patients compared to controls

^d^FDR = False discovery rate

### Genome wide region level analysis identifies hundreds of differentially methylated loci in FCX and CER

**Fig. 2:**
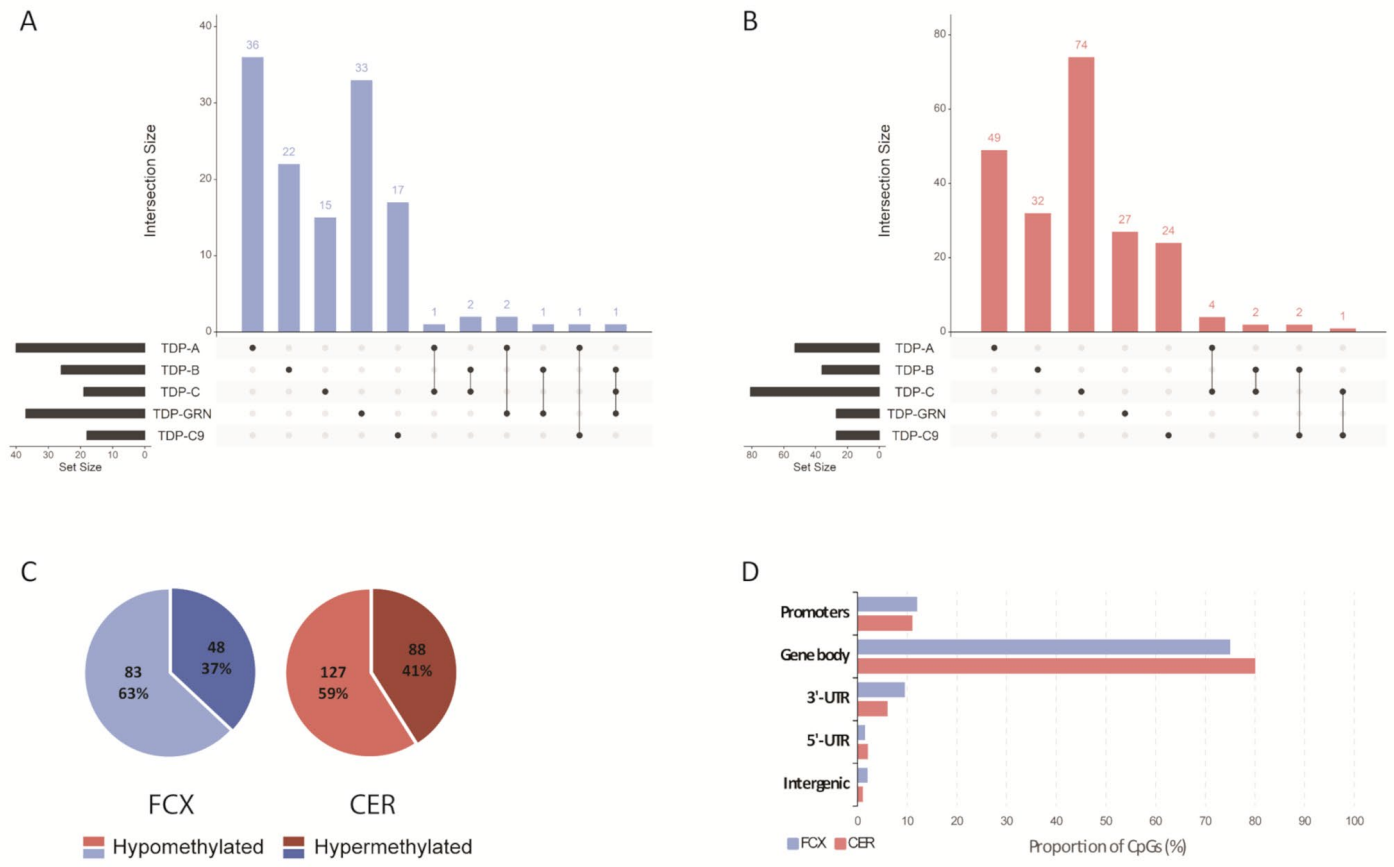
RRBS identifies hundreds of DMRs in brain tissue from FLTD-TDP patients. Upset plot showing the number of unique and overlapping DMRs in each pathological group, in FCX (**A**) and CER (**B**). Distribution of hypomethylated (light shades) and hypermethylated (dark shades) DMRs across all groups, in FCX (left; blue graph) and CER (right; red graph) (**C**). Proportion of DMRs in the context of its position relative to the annotated genes. Proportions are shown for both FCX (blue bars) and CER (red bars) DMRs across all groups (**D**)

Next, we expanded our analyses beyond promoters to genome wide level, while still performing group comparisons in each brain region separately. From these analyses we identified hundreds of differentially methylated DMRs, with a total of 131 in FCX and 215 in CER across all patient groups, annotated to 123 and 203 genes, respectively (Fig. 2A and B; Supp. Fig. 4A and B; Supp. Tables 4 and 5). Of these, we found a similar proportion of hyper- and hypomethylated loci in both tissues, with most loci being hypomethylated (Fig. 2C; Supp. Tables 4 and 5). Regarding the genomic context of these loci in both tissues, the overwhelming majority was located within a gene body (75% in FCX and 80% in CER), followed by gene promoters (12% in FCX and 11% in CER), 3’-UTRs (9.5% in FCX and 6% in CER), and a small proportion in intergenic regions (2% in FCX and 1% in CER) and within 5’-UTRs (1.5% in FCX and 2% in CER; Fig. 2D; Supp. Tables 4 and 5). Akin to our findings from the CpG-level analyses, most DMRs are unique to pathological subtypes and thus, combining patient subgroups for analysis only contributed a limited amount of additional DMRs with three in FCX (annotated to *PSMA6* in group ABC, and to *NDUFA10* and *SEMA3C* in group TDP) and four in CER (annotated to *FHL2*, *PDGFRA*, and *BLCAP* in group ABC, and *DHDDS* in group TDP). In FCX, the strongest finding overall was a hypomethylated gene body DMR within *GFPT2* (which spans exons 14 and part of the adjacent introns) in several group comparisons (TDP-B, TDP-C, TDP-GRN, group ABC, and group TDP; Supp. Table 4). Interestingly, and although not as strong as in FCX, *GFPT2* is one of only five genes where DMRs were found in both FCX and CER (TDP-B; Table 5). We selected this locus to validate our RRBS finding, focusing on TDP-C which showed the strongest effect (logFC= -2.27; FDR = 1.2E-03; Supp. Figure 4C). We selected one highly methylated sample (> 80% methylation), one lowly methylated sample (< 20% methylation), as well as two samples with intermediate methylation per group (*N* = 4 TDP-C and *N* = 4 neuropathologically normal controls) based on methylation values across the region, measured by RRBS. Bisulfite sequencing (BS) targeted to the *GFPT2* DMR showed at most a 10% difference in methylation level (range 1–10%) as compared to RRBS, with none of the samples changing their categorical classification of high/intermediate/low methylation, providing support and validation to our RRBS findings (Supp. Figure 4D).

**Table 5:** Genes harbouring DMRs in both frontal cortex and cerebellum

Brain region	Genomic position^a^	Annotated gene^b^	Genomic context	Group	logFC^c^	FDR^d^
FCX	chr1:3,168,538–3,168,584	*PRDM16*	Gene body	TDP-A	-1.1151	8.36E-03
				TDP-C	-1.4589	1.34E-03
	chr1:3,311,222–3,311,248			TDP-GRN	-1.4791	0.04262
	chr4:54,094,020–54,094,059	*PDGFRA*	Gene body	TDP-C9	1.3602	0.02829
	chr5:180,313,751–180,314,251	*GFPT2*	Gene body	TDP-B	-1.4467	0.03312
				TDP-C	-2.2726	1.2E-03
				TDP-GRN	-1.6770	0.03083
				ABC	-1.4365	2.28E-04
				TDP	-1.1243	5.46E-03
	chr17:16,746,074–16,746,110	*CCDC144A*	Gene body	TDP-C	-1.0961	0.01089
				ABC	-0.9831	1.56E-03
	chr19:16,355,251–16,355,751	*EPS15L1*	3’-UTR	TDP-C	0.9933	2.81E-03
CER	chr1:3,426,223–3,426,278	*PRDM16*	Gene body	TDP-GRN	-1.7168	7.2E-03
	chr4:54,093,251–54,093,751	*PDGFRA*	Gene body	ABC	-0.9642	0.03194
	chr5:180,313,501–180,314,001	*GFPT2*	Gene body	TDP-B	-1.8570	0.02139
	chr17:16,746,074–16,746,110	*CCDC144A*	Gene body	TDP-C	-0.8375	0.04997
	chr19:16,363,001–16,363,501	*EPS15L1*	Gene body	TDP-A	-0.87425	0.03796

^a^GRCh38 assembly

^b^Nearest gene according to GENCODE v44

^c^FC = Fold change. Negative and positive values denote hypo- and hypermethylation, respectively, in patients compared to controls

^d^FDR = False discovery rate

Additionally, between the two DMR analyses (promoter and genome-wide), we identified only three loci in common, with one in FCX (overlapping *PARVG*/*PARVB*; Table 3 and Supp. Table 4), and two in CER (overlapping *DHX33*/*DHX33-DT* and a known CpG island within *OTX2*/*OTX2-AS1*; Table 4 and Supp. Table 5).

Finally, we investigated whether an impaired epigenetic machinery could represent a potential mechanism underlying the widespread DNA methylation changes we observed in FTLD-TDP patients. Using our previously generated bulk RNA sequencing dataset [10] we assessed expression levels of a subset of genes encoding for DNA methylation ‘writers’ or methyltransferase enzymes (*DNMT1* responsible for methylation maintenance, and *DNMT3A/B* responsible for *de novo* methylation), as well as DNA methylation ‘erasers’ (*TET1*, *TET2* and *TET3*, which are key players in the first step of the demethylation process), in FTLD-TDP patients and neuropathologically normal controls. Results from these analyses highlight expression changes in FCX in genes from both groups of DNA methylation regulators, namely *DNMT1* (higher in FTLD-TDP; *P* = 4E-03) and *TET3* (lower in FTLD-TDP: *P* = 2.7E-05), whereas in CER we found changes in *TET1* (lower in FTLD-TDP; *P* = 1.3E-02) (Supp Fig. 5A). Furthermore, besides global changes across all FTLD-TDP patients, we also observed specific expression patterns of the assessed genes to some pathological subtypes (Supp Fig. 5B), suggesting that to some extent, differential expression of epigenetic machinery components may contribute to the methylation changes we observe with both pathological subtype and brain region specificity.

### Enrichment analysis identifies distinct processes in TDP pathological subtypes

To gain insight into potential underlying functions or pathways in genetically unexplained FTLD-TDP patients (sporadic patient groups TDP-A, TDP-B, TDP-C and combined ‘ABC’) where we identified the most changes, we next performed Gene Ontology (GO) analyses focusing on the “Biological Process” (BP) and “Molecular Function” (MF) categories and using the differentially methylated genes from all analysis in each pathological group as input in FCX and CER separately (Supp. Tables 6 and 7). In the BP category, we identified 53 clusters of related terms in FCX and 52 in CER. In the MF category, we identified substantially less clusters with seven in FCX and eight in CER (Supp. Tables 6 and 7).

**Fig. 3:**
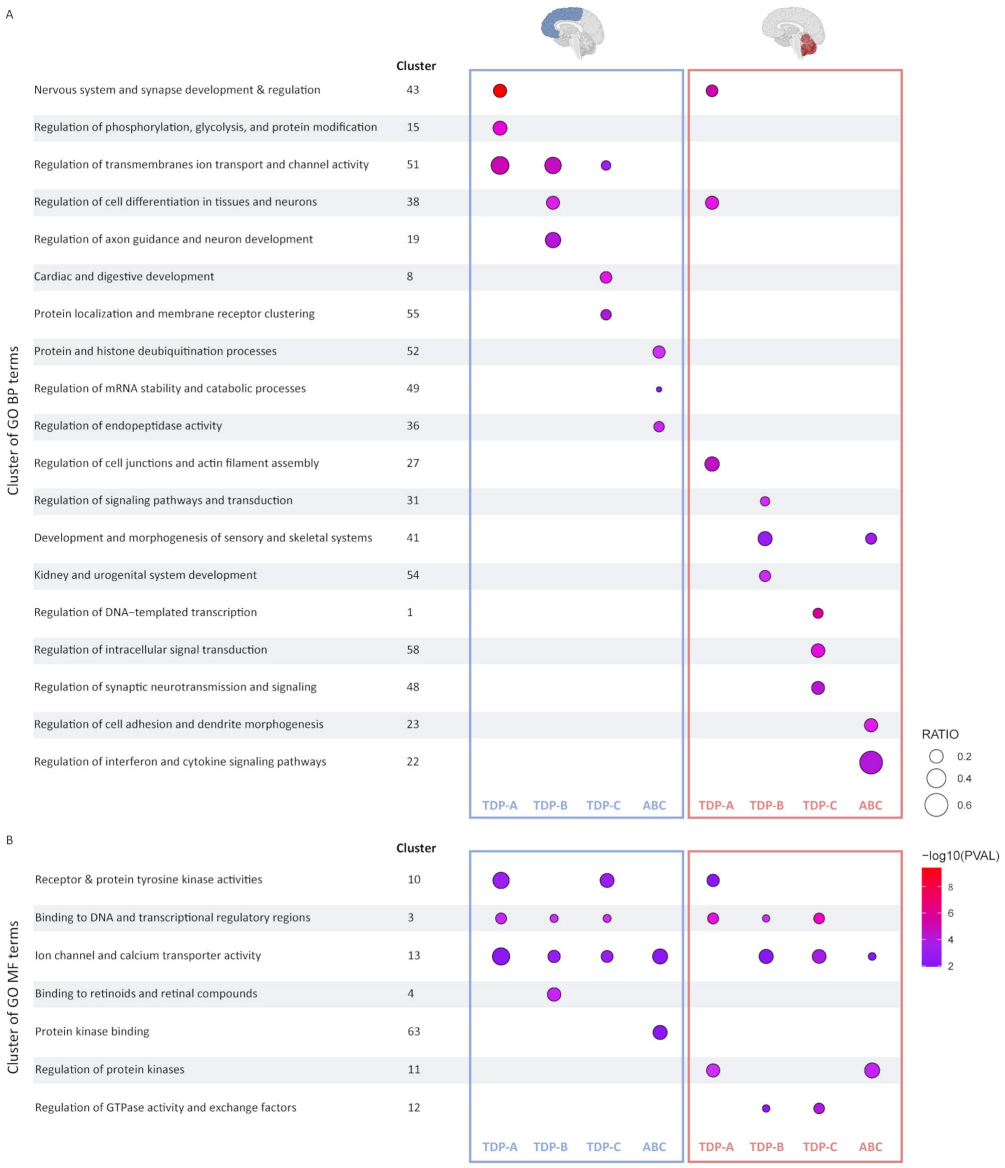
Top 3 clusters of Gene Ontology terms enriched in FTLD-TDP pathological groups. Clusters of GO terms significantly enriched in each sporadic pathological group in FCX (left; blue boxes) and CER (right; red boxes) from the biological process (**A**) and molecular function (**B**) categories. Results are shown for the most significant enriched terms in the top 3 clusters from each group, with circle color representing Pvalue and circle size representing the gene ratio in the term

In the BP category, although we observed overall a large overlap of identified clusters (several related enriched terms that cluster together; Supp. Table 6), the top 3 processes are largely non-overlapping between pathological subtypes as well as tissue types (Fig. 3A). In TDP-A, terms related to nervous system and synapse development and regulation were the most significant in both FCX and CER (cluster 43; top GO term “Nervous system development”; 3.82E-10 in FCX and 7.11E-06 in CER). We further detect enrichment in FCX for terms related to regulation of phosphorylation, glycolysis, and protein modification (cluster 15; top GO term “Protein autophosphorylation”; *P* = 4.29E-06). Of note, and albeit not in the top 3, we identified two clusters that are not only unique to FCX but also to a specific pathological subtype. These included cluster 2 in TDP-A including terms related to DNA damage repair (top GO term “Recombinational repair”, *P* = 0.039), and cluster 37 in TDP-B including terms related to cholesterol biosynthesis (top GO term “Regulation of cholesterol biosynthetic process”, *P* = 0.011) (Supp. Table 6; Supp Fig. 6). In CER from TDP-B, we found the strongest enrichment in terms related to regulation of signaling pathways and transduction (cluster 31, top Go term “Regulation of signal transduction”; *P* = 6.64E-04). In TDP-C, we found an enrichment in terms related to protein localization and membrane receptor clustering in FCX (cluster 55; top GO term “Protein localization to membrane”; *P* = 1.01E-04), and to regulation of DNA-templated transcription in CER (cluster 1; top GO term “Positive regulation of transcription by RNA Polymerase II”; *P* = 2.27E-06). Across all groups in FCX, terms related to ion transport were highly enriched (cluster 51), whereas in the combined ABC group, we detected the strongest enrichment in terms related to protein and histone deubiquitination processes (cluster 52; top GO term “Protein K48-linked deubiquitination”; *P* = 3.25E-04).

Finally, in the MF category, we observed a large overlap of enriched clusters between pathological subtypes and across tissues (Supp. Tables 6 and 7). Importantly, we found two clusters in common between all TDP subtypes in both brain regions, namely terms related to binding to DNA and transcriptional regulatory regions (cluster 3), as well as ion channel and calcium transporter activity (cluster 13) (Fig. 3B; Supp Fig. 6).

### Methylation levels at several DMRs correlate with gene expression levels

**Fig. 4:**
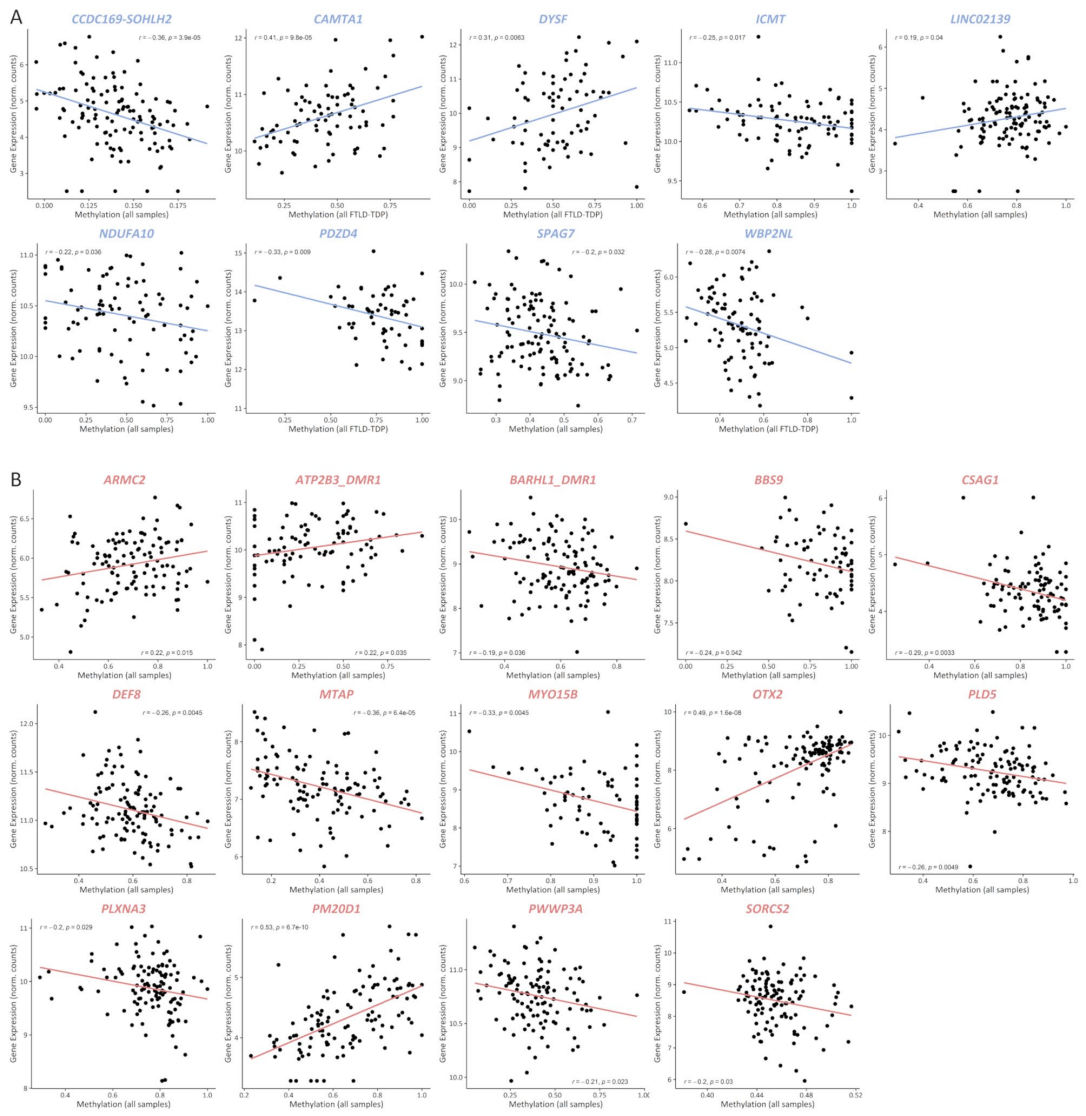
DMR methylation levels correlate with expression of annotated genes. Pearson correlation between DMR methylation and expression levels of the annotated genes for 9 genes in FCX (**A**) and 14 genes in CER (**B**). Only significant correlations are shown, and plotted are the strongest correlations for each gene, either including controls (all samples) or only FTLD-TDP patients (all FTLD-TDP) as indicated in the X-axis (see also Supp. Table 8)

**Fig. 5:**
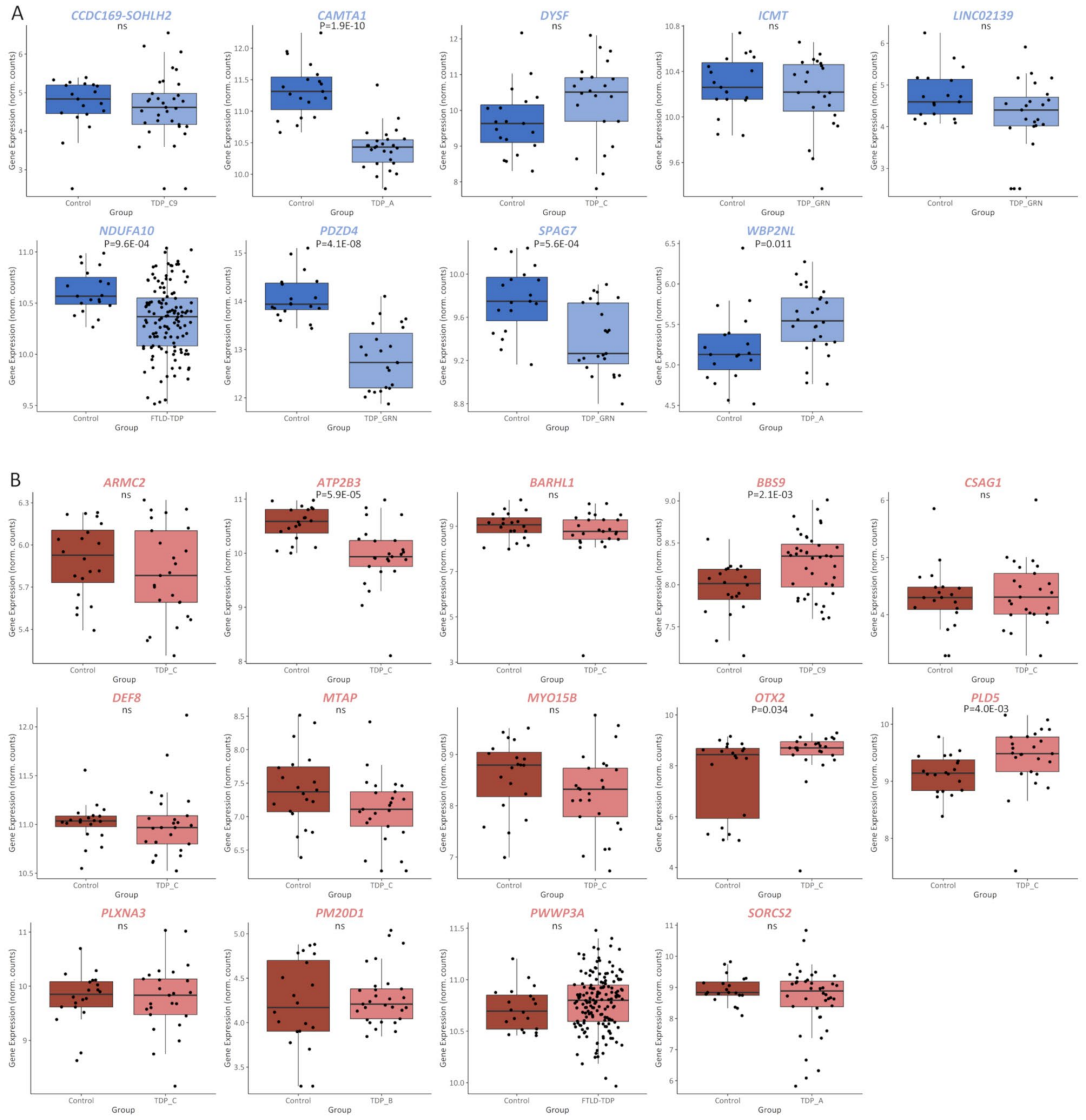
DMR containing genes are differentially expressed. Gene expression of all genes for which expression correlates with methylation levels in FCX (**A**) and CER (**B**). Comparisons are shown for expression levels of the annotated gene between controls and the pathological group in which the DMR was identified, as indicated in the X-axis. Pvalue from each comparison is shown, with ns = not significant

Given that altered gene expression is the most common and well-studied consequence of aberrant methylation, we next interrogated our previously generated bulk brain transcriptomic dataset [10] to assess correlations between methylation levels within all DMRs (from both promoter and genome-wide analyses) and the expression of the associated gene(s) for which expression was measured in FCX or CER. When several overlapping DMRs were identified within the same gene, they were merged into one single DMR with the coordinates of the largest region, whereas if several non-overlapping DMRs were identified within the same gene, they were treated as independent DMRs with correlations calculated for each. To increase statistical power, correlations were calculated including all study individuals (ALL; FTLD-TDP and controls combined) (Fig. 4; Supp. Tables 8 and 9). We found correlations between methylation and expression of the annotated gene for nine DMRs in FCX (*CCDC169-SOHLH2*, *CAMTA1*, *DYSF*, *ICMT*, *LINC02139*, *NDUFA10*, *PDZD4*, *SPAG7* and *WBP2NL*; Fig. 4A) and 14 in CER (*ARMC2*, *ATP2B3*, *BARHL1*, *BBS9*, *CSAG1*, *DEF8*, *MTAP*, *MYO15B*, *OTX2*, *PLD5*, *PLXNA3*, *PM20D1*, *PWWP3A* and *SORCS2*; Fig. 4B). Interestingly, for four genes in FCX, we found that the correlations became stronger when including only FTLD-TDP patients, namely *CAMTA1*, *PDZD4*, *WBP2NL*, and *DYSF*, suggesting that disease environment may play a role in the methylation effect (Supp. Table 8). Next, for each of the 23 genes, we investigated whether differential expression was observed in the pathological subtypes where the DMR was identified, which was the case for nine genes: (**i**) five in FCX, namely *CAMTA1* (lower expression in the TDP-A group; *P* = 1.9E-10); *PDZD4* (lower expression in the TDP-GRN group; *P* = 4.12E-08); *SPAG7* (lower expression in the TDP-GRN group; *P* = 5.6E-04); *NDUFA10* (lower expression in all FTLD-TDP combined; *P* = 9.6E-04); and *WBP2NL* (higher expression in the TDP-A group; *P* = 0.011) (Fig. 5A); and (**ii**) four in CER, with three in the TDP-C group, namely *ATP2B3* (lower expression in TDP-C; *P* = 5.9E-05); *PLD5* and *OTX2* (higher expression in TDP-C; *P* = 4.0E-03 and *P* = 0.034, respectively), and *BBS9* in the TDP-C9 group (higher expression in TDP-C9; *P* = 2.1E-03) (Fig. 5B). No differential expression was observed for the other genes within the groups where the DMR was identified, compared to controls. In addition, for some genes we observed differential expression in pathological subtypes beyond those where the DMR was identified (Supp. Figure 7), suggesting that additional factors besides DNA methylation may modulate the expression of these genes. One such factor could be altered expression of epigenetic machinery components that regulate transcription via epigenetic modulation. To explore this hypothesis, we investigated whether the expression of a subset of genes encoding for methyl-CpG binding proteins (MBPs; namely *MBD1*, *MBD2*, *MBD3* and *MECP2*), which bind to methylated DNA and recruit additional factors to modulate gene expression, was altered in FLTD-TDP patients. Results from these analyses show that in FTLD-TDP patients, *MBD2* expression is increased in both FCX and CER (*P* = 1.6E-02 and *P* = 3.6E-03, respectively), as well as *MBD3* in CER (*P* = 4.7E-03), as compared to neuropathologically normal controls (Supp Fig. 8), suggesting that differential expression of such components may play a role in the limited correlation between differentially methylated genes and their expression.

### *CAMTA1* expression is mediated by both methylation changes and TDP-43 levels

**Fig. 6:**
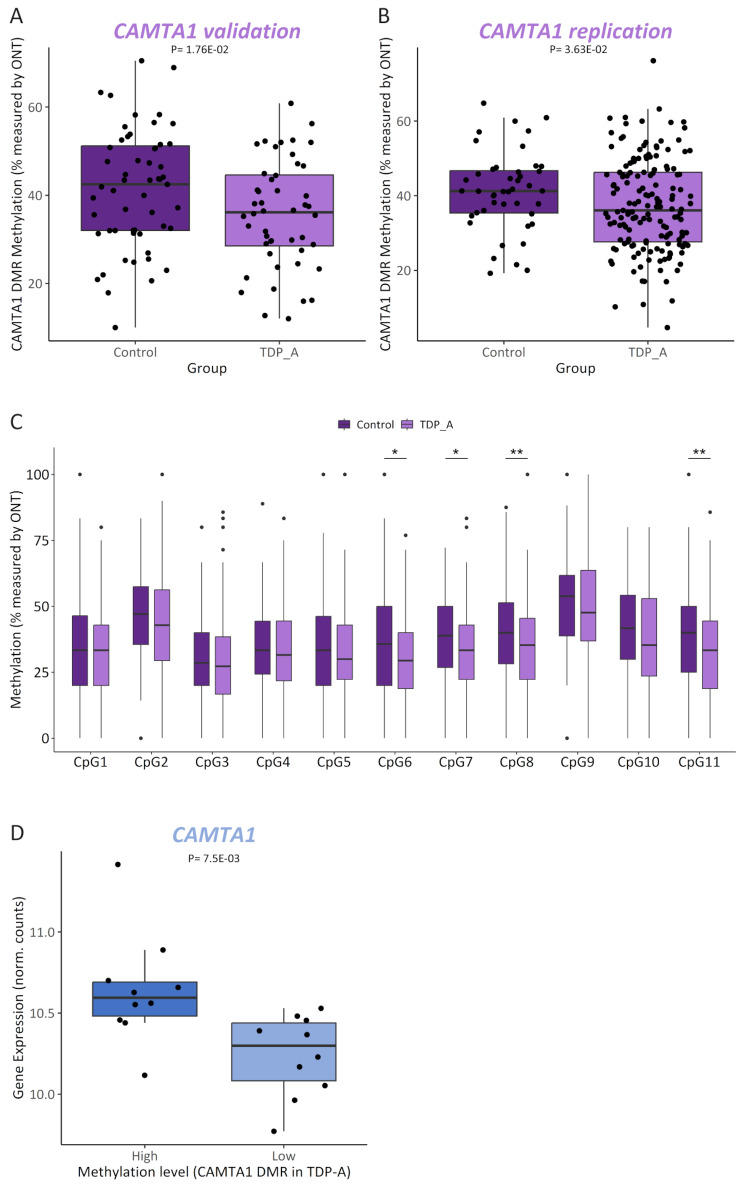
*CAMTA1* is differentially methylated in TDP-A. Methylation levels measured by ONT long-read sequencing in FCX from controls (*N* = 28) and TDP-A (*N* = 25) overlapping with the RRBS study (*CAMTA1* validation) (**A**) or in an independent replication cohort of controls (*N* = 22) and TDP-A (*N* = 80) (**B**). Plotted are both haplotypes from each sample and the adjusted Pvalue from each comparison is shown. Methylation levels measured by ONT long-read sequencing in the full cohort (combined validation and replication) of controls (dark shade boxes) and TDP-A (light shade boxes) at each CpG profiled within the *CAMTA1* DMR. Wilcoxon signed-rank test with *P < 0.05 and **P < 0.01 (**C**). *CAMTA1* expression levels in TDP-A patients (*N* = 20) stratified by methylation levels (*N* = 10 highest and *N* = 10 lowest samples; dark and light shades, respectively) as measured by RRBS

Its pivotal role in several processes such as regulating long-term memory [77] as well as neuronal development, maturation and survival [78], together with evidence of being a TDP-43 target [11, 79–81], made *CAMTA1* an especially interesting and relevant finding in the context of FTLD-TDP pathology. As such, we selected this locus for further follow up. A closer inspection of the 185 bp *CAMTA1* DMR revealed that it is located within intron 6 of *CAMTA1* (NM_015215) in chromosome 1p36 (Supp Fig. 9A), and harbors several hypomethylated CpGs in the TDP-A group compared to controls (Supp. Figure 9B). First, to validate our *CAMTA1* DMR finding, we investigated whether we could detect differential methylation at the *CAMTA1* DMR, measured with an alternative technique to RRBS. For this, FCX DNA samples from TDP-A (*N* = 25) and control (*N* = 28) individuals overlapping with the RRBS study, were sequenced using ONT long-read sequencing, which also profiles CpG methylation. With ONT long-read sequencing we also confirmed the lower methylation levels in the TDP-A group compared to controls (logFC = -0.366; *P* = 0.0176; Fig. 6A). Next, also using ONT long-read sequencing, we sought to replicate this finding using an independent cohort of TDP-A (*N* = 80) and control (*N* = 22) samples, which corroborated the finding showing a hypomethylated DMR in TDP-A patients compared to controls (logFC = -0.276; *P* = 0.0363) (Fig. 6B). When combining the discovery and replication cohorts, a similar effect was observed (logFC = -0.27; *P* = 3.76E-03; Supp Fig. 9C). Next, using ONT sequencing data in the full cohort, we analyzed individual CpG sites within the *CAMTA1* DMR to determine the most relevant CpGs driving the hypomethylation signal. We observed lower methylation in the TDP-A group at all CpGs measured in the locus, with CpG numbers 6, 7, 8 and 11 showing the strongest effect (Fig. 6C), suggesting that these sites have the highest predictive value as proxy for the methylation levels within the region. Finally, to confirm previous reports of *CAMTA1* being a TDP-43 target, we used an additional transcriptomic dataset from *TARDBP* KD hiPSC-derived cortical neurons [50], which revealed a positive correlation between the expression of *CAMTA1* and *TARDBP* genes, albeit just below significance using the limited data points available (*r* = 0.74, *P* = 0.057; Supp. Figure 9D), suggesting that *CAMTA1* is indeed a TDP-43 target. To disentangle the relationship between the effects of TDP-43 dysfunction and methylation on the levels of *CAMTA1*, we next compared *CAMTA1* levels within the group of TDP-A patients using stratification by methylation level, based on RRBS values across the *CAMTA1* DMR (*N* = 20; comparing 10 samples with the highest methylation to 10 samples with the lowest methylation levels). This again showed lower *CAMTA1* expression in the lower methylation group compared to the higher methylation group (*P* = 7.5E-03; Fig. 6D), suggesting that methylation changes at this DMR affect *CAMTA1* expression independently and cumulatively to TDP-43 dysfunction.

### Aberrant methylation at the *CAMTA1* DMR alters expression of additional genes in the 1p36 locus

**Fig. 7:**
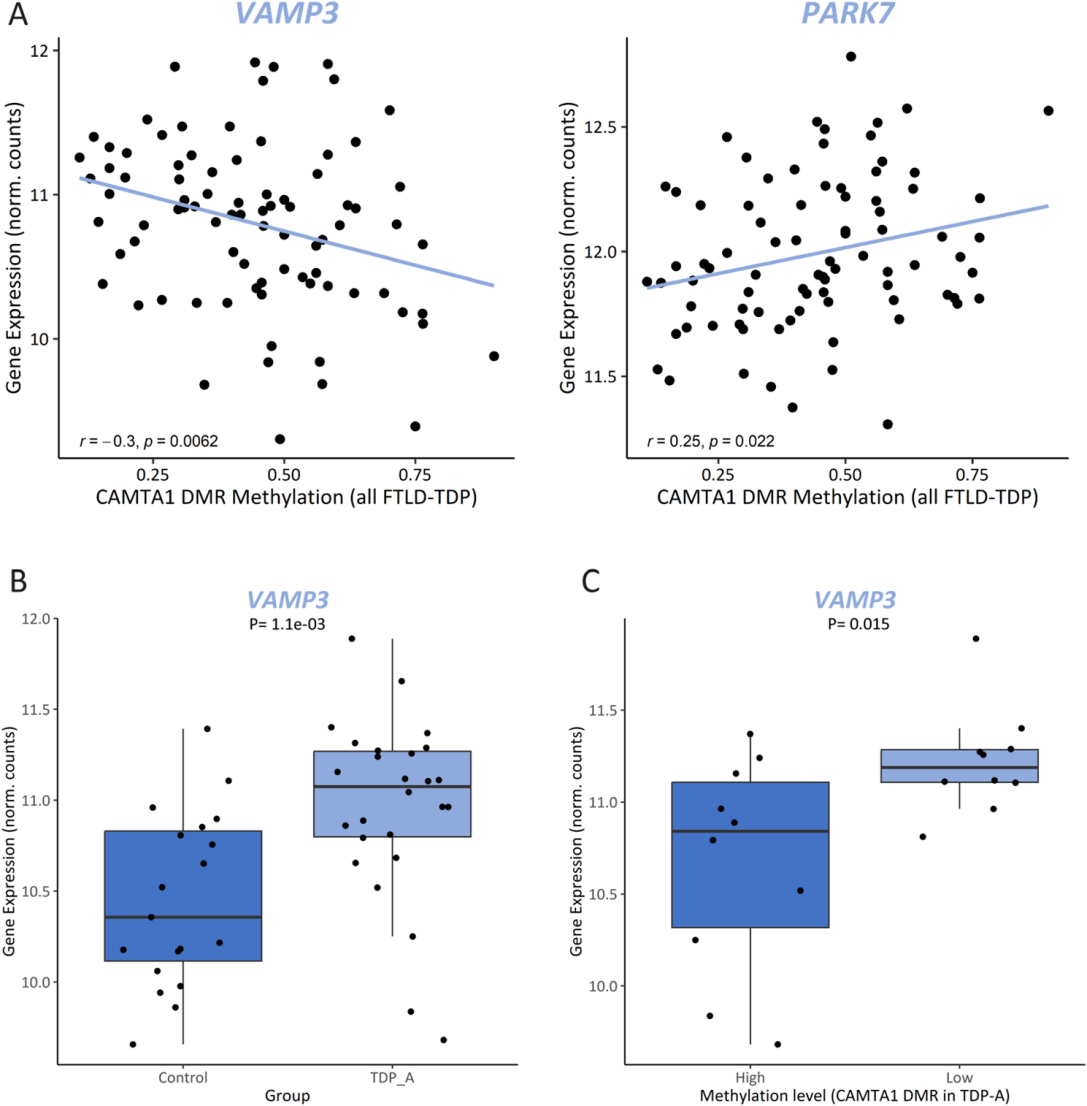
Methylation changes at the *CAMTA1* DMR alters expression of additional genes in the locus. Pearson correlation between methylation levels at the *CAMTA1* DMR and the expression levels of *VAMP3* (left panel) and *PARK7* (right panel) in FCX from FTLD-TDP patients (**A**). *VAMP3* expression levels in FCX from controls and TDP-A (**B**) and only in TDP-A patients (*N* = 20) stratified by methylation levels (*N* = 10 highest and *N* = 10 lowest samples; dark and light shades, respectively) as measured by RRBS (**C**)

**Fig. 8:**
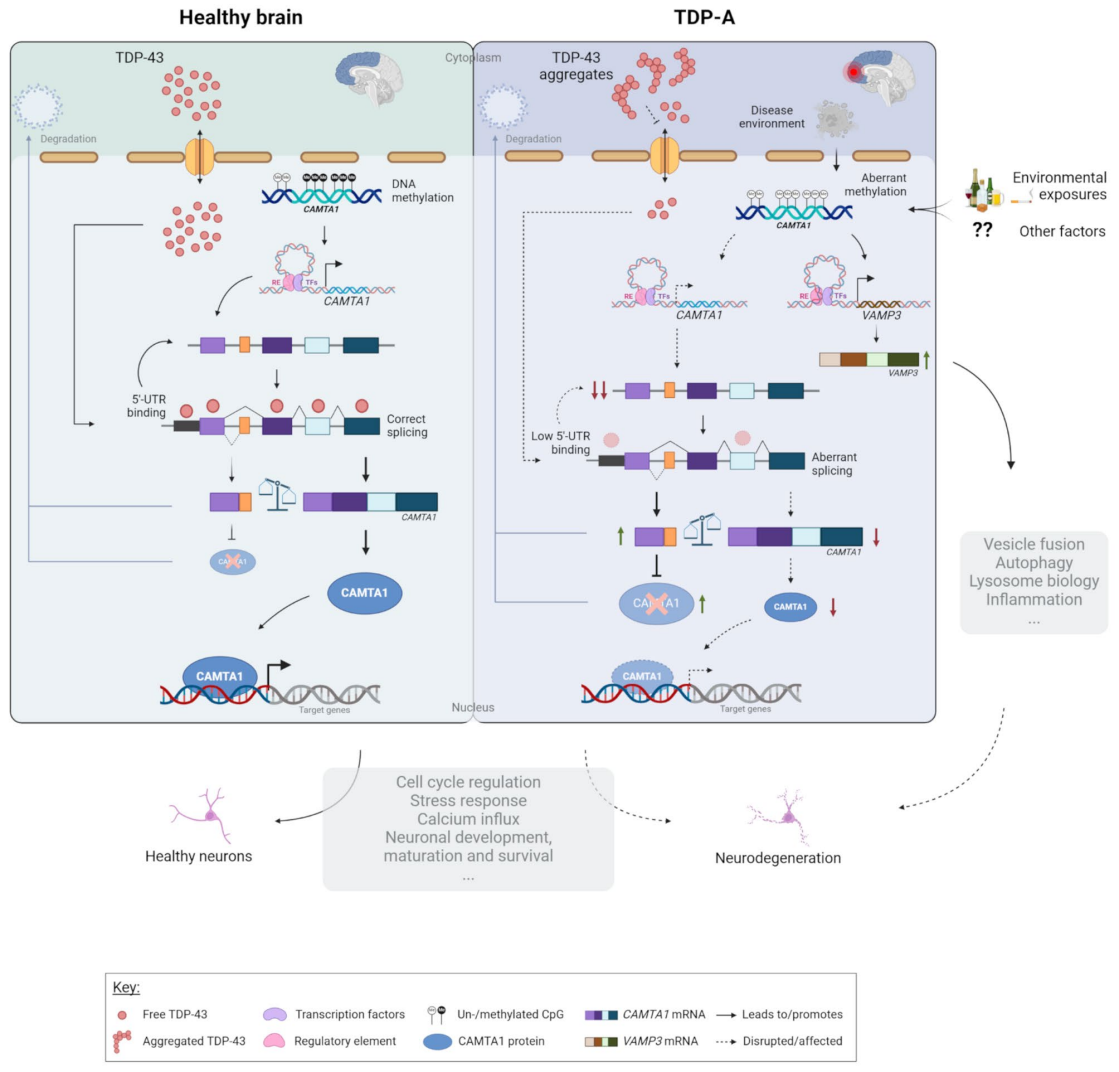
Proposed *CAMTA1* double-hit model. In normal physiological conditions, TDP-43 is shuttled between the cytoplasm and the nucleus where it exerts its function. Once in the nucleus, TDP-43 ensures correct splicing of *CAMTA1* and enhances *CAMTA1* expression through direct binding to the 5’-UTR. Physiological levels of *CAMTA1* are thus maintained by proper TDP-43 function and normal *CAMTA1* methylation. In FTLD-TDP brains, as a consequence of TDP-43 aggregation, TDP-43 is less available in the nucleus and no longer ensures proper *CAMTA1* splicing and/or binding to its 5’-UTR, thereby reducing *CAMTA1* expression. In addition, and independently from TDP-43 dysfunction in TDP-A patients, due to a combination of factors such as disease environment and/or environmental exposures, methylation within the *CAMTA1* gene body is lost. Hypomethylation in this region affects the expression of *CAMTA1* and additional genes in the locus such as *VAMP3*, possibly through altering chromatin conformation and/or transcription factor binding, which in turn modulates the function of regulatory elements in the locus. As a transcriptional activator of several target genes, CAMTA1 is involved in a multitude of processes that are critical for neuronal health. Impairment of such CAMTA1-dependent mechanisms in a double-hit fashion produced by both nuclear TDP-43 and CAMTA1 methylation levels, together with alterations in processes regulated by VAMP3, may contribute to neurodegeneration and the pathology observed in TDP-A patients

Mining the UCSC Genome Browser [82] revealed that this intronic DMR, which is not within a known CpG island, overlaps with an open chromatin region (defined by the DNaseI hypersensitivity clusters track from ENCODE V3), as well as several transcription factor binding sites (defined by the Transcription factor ChiP-seq clusters track from ENCODE V3), suggesting a high regulatory potential (Supp. Figure 10). Analyzing additional datasets aimed at profiling genome-wide regulatory elements (Roadmap Epigenomics [83], GeneHancer [84]) further revealed that the DMR overlaps an enhancer element (GH01J006404; GeneHancer) of which *CAMTA1* is a predicted target (Supp. Figure 10). Broadening the analysis to the intron that harbors the DMR revealed a region rich in enhancer elements predicted to target several genes within the locus. Specifically in brain tissue [85], evidence supports the existence of enhancer elements in several brain regions predicted to target the neighboring gene *VAMP3* (Supp. Figure 10). Given that methylation changes may alter chromatin conformation and thus affect the functioning of regulatory elements, we investigated whether aberrant methylation at the *CAMTA1* DMR alters the expression of additional genes in the locus, besides *CAMTA1*. Testing all genes within 1 MB from the DMR, we found that methylation levels within the region correlate with the expression of *VAMP3* (r_TDP_ = -0.3, P_TDP_ = 6.2E-03) and *PARK7* (r_TDP_=0.25, P_TDP_=0.022) in FCX; however, only within TDP patients (Supp Table 10; Fig. 7A). When comparing TDP-A to controls, we found that only *VAMP3* is differentially expressed in FCX (increased in the TDP-A group; *P* = 1.1E-03; Fig. 7B; Supp Fig. 11A) and that expression changes are also observed in additional pathological groups (Supp. Figure 11B). Furthermore, when investigating the effect of methylation on gene expression, within TDP-A patients stratified by methylation levels, we found that *VAMP3* is differentially expressed between the two groups, with higher *VAMP3* expression in the low methylation group (*P* = 0.015; Fig. 7C). Finally, querying the CLIPdb module of the POSTAR3 database [81] revealed no TDP-43 binding sites within *VAMP3* in brain tissue, which is corroborated by our own transcriptomic dataset from *TARDBP* KD neurons (Supp. Figure 11C), suggesting that *VAMP3* is not a TDP-43 target and that expression changes might be, at least in part, modulated by methylation changes at the *CAMTA1* DMR. Taking ours and others’ findings together, we propose a working model for the *CAMTA1* DMR and locus where on the one hand, in healthy brains, *CAMTA1* levels are maintained both via nuclear TDP-43 (i.e. promoting adequate *CAMTA1* splicing and expression through direct binding to the 5’-UTR), as well as correct gene body methylation. On the other hand, aggregation and subsequent accumulation of TDP-43 in the cytoplasm leads to TDP-43 loss-of-function and lower TDP-43-dependent *CAMTA1* levels. In addition, and independently from TDP-43 dysfunction in TDP-A patients, hypomethylation within the *CAMTA1* gene body alters chromatin availability and/or function of regulatory elements in the locus, further reducing *CAMTA1* expression while activating nearby genes such as *VAMP3*. Dysfunction of both CAMTA1- and VAMP3-dependent mechanisms may contribute to neurodegeneration and the pathology observed in TDP-A patients. (Fig. 8).

## Discussion

In this study, we present the most extensive analysis of the DNA methylation landscape of FTLD patients with TDP-43 pathology performed to date and provide a unique resource dataset for the community. By including patients presenting with different pathological subtypes, we aimed at profiling epigenetic changes across the full spectrum of disease, from sporadic to genetic forms of FTLD. In addition to the large cohort of samples, we also profiled two brain regions from each individual. To investigate the strongest and most disease relevant changes, we included the frontal cortex, which is the brain region where TDP-43 pathology and dysfunction is highest. To investigate potential changes at earlier stages of disease, we also profiled the cerebellum, a relatively spared region from pathology but with increasing evidence supporting a strong involvement in the disease process [11, 86, 87]. To measure DNA methylation in each sample, we employed RRBS and analyzed differential methylation both at the single CpG site level as well as within regions or clusters of CpG sites.

When comparing FTLD-TDP to controls, we identified thousands of differentially methylated CpGs across all pathological subgroups, highlighting the potential for epigenetics to play a role in FTLD disease mechanisms. Interestingly, although we identified methylation changes across all patients, we observed that most of our findings are unique and specific to pathological and genetic subtypes, with less than 10% of all CpGs being shared between patient groups. Although we cannot exclude the potential for false positive or negative signals due to our relatively small sample sizes, our results suggest that FTLD subtypes not only have distinct transcriptomic [10] and genetic [73] signatures as previously proposed, but are also distinct at the epigenetic level. In addition, we also found that the majority of methylation changes that we identified are brain region-specific with only a small percentage of overlap between frontal cortex and cerebellum, an observation that is in line with the previous finding that the overlap of transcriptomic changes between cortical tissues and cerebellum is lower than between different cortical tissues [87].

Within FTLD genes, we identified the highest number of differentially methylated CpGs within *NFATC1*, a gene implicated in disease through a recent DNA methylation study [29], in which the authors identified a hypermethylated gene-body CpG in FTLD patients compared to controls. We identified a different set of differentially methylated CpGs, including one in the 5’UTR which was correlated with *NFATC1* expression, thus suggesting that several sites may contribute to the altered *NFATC1* expression observed in FCX of FTLD-TDP patients.

Similar to our results from the CpG level analysis, we also found that the majority of DMRs are unique to a pathological subtype as well as brain region, regardless of the genomic context of the DMR. Interestingly, we identify a large proportion of DMRs in sporadic patient groups (TDP-A, TDP-B and TDP-C) across both tissues, suggesting that the contribution of epigenetic changes may be more relevant in these patients. In fact, gene ontology enrichment analyses revealed several processes possibly contributing to the etiology of different pathological subtypes, as well as potential underlying disease mechanisms common across subtypes. In FCX, affected biological processes included protein autophosphorylation and DNA damage repair in TDP-A; cholesterol biosynthesis in TDP-B; and protein localization in TDP-C. We further detected shared processes, such as regulation of transmembrane ion transport and channel activity across all subtypes, and protein deubiquitination in ABC. Importantly, all these processes are highly relevant in FTLD. As part of the protein autophosphorylation cluster, we found several tyrosine kinases and receptor tyrosine kinases to be differentially methylated in TDP-A. Among others, we identified *ALK* and *LTK*, which encode for two closely related receptor tyrosine kinases with important roles both at the developmental stage as well as in adult brain. Animal models showed that absence of Ltk and Alk leads to impaired neuronal migration and cortical patterning in the developing cortex, and aberrant neuronal projections and axon tracts in adult cortices, leading to behavior abnormalities and impaired cognitive functions [88, 89]. Interestingly, these effects are mediated through the activation of the insulin-like growth factor 1 receptor, encoded by *IGFR1*, which we also find differentially methylated in TDP-A [88]. In addition, we found neurotrophic tyrosine kinase receptor 1 (*NTRK1*), which is also implicated in neuronal damage, cognitive impairment and behavioral changes [90, 91]. Importantly, among the genes in this cluster, we find several that are associated with behavioral abnormalities, a clinical hallmark of TDP-A patients [92]. Also in these patients, we identified an enrichment in terms related to DNA damage repair, a process whose dysfunction has been extensively implicated in neurodegenerative diseases including ALS/FTD [93–95]. In our analysis we identify several genes involved in double strand DNA damage repair by homologous recombination, such as *RAD50*, one of the components of the MRE11-RAD50-NBS1 complex that is initially recruited to the break site to mediate DNA end resection. The resulting exposed ssDNA tails are then coated with RAD51 monomers, a process that is dependent on several mediators such as *BRCA1*, *RAD52*, and the RAD51 paralogs *RAD51B*, *RAD51D* and *XRCC3* [96, 97], all of which we also found to be differentially methylated. *RAD52* also plays an additional important role in regulating a repair pathway through single strand annealing, when the break site contains repeated DNA sequences [96]. Combined dysfunction of such key players could profoundly impact DNA damage repair, promoting persistent genome damage that leads to neurodegeneration. Finally, a previous study reported an enrichment of splicing and transcript-level changes in genes involved in DNA repair in human brain, and showed that these changes are age-related rather than due to neuronal loss associated with neurodegeneration [98]. The fact that we detect an enrichment in these terms specifically in TDP-A may reflect in part the fact that these patients are overall older; however, our cohort of neuropathologically normal controls is the oldest, suggesting that the disease environment may accelerate the dysfunction of this process. In TDP-B, we found terms related to cholesterol biosynthesis to be specifically affected in these patients and brain region. Driving this enrichment are both sterol regulatory element-binding protein coding genes, *SREBF1* which mainly regulates fatty-acid synthesis, and *SREBF2* which is responsible for cholesterol homeostasis [99]. In the central nervous system, lipids and lipid metabolism are critical for its development and function, with reports of disrupted homeostasis in several neurodegenerative diseases. Importantly, studies have reported that TDP-43 depletion leads to reduced *SREBF2* expression by directly binding to its mRNA leading to the disruption of cholesterol metabolism, which in turn affects myelination [100, 101]. Furthermore, defective cholesterol metabolism has been extensively associated with amyotrophic lateral sclerosis [101–103], the most common concomitant disorder of FTLD-TDP and specifically TDP-B patients, where we detected this enrichment.

In CER, we identified nervous system and synapse development to be affected in TDP-A, and signal transduction in TDP-B, whereas transcription regulation was mostly affected in TDP-C. Finally, we found terms related to DNA binding and transcription factor activity to be highly enriched across all subtypes and brain regions, which were also recently found in FCX from FTLD patients [29]. Importantly, we now provide evidence for these enriched terms not only in FCX but also in CER from FTLD patients, with distinct genes driving the enrichment in each tissue. On the one hand, in FCX we find *CAMTA1* and general transcription regulators, including genes that encode for subunits 3 and 5 (*GTF3C3* and *GTF3C5*, respectively) of the DNA-binding transcription factor TFIIIC complex, which plays a critical role in the recruitment of RNA Polymerase III (Pol III) [104]. Pathogenic variants in Pol III cause a type of hypomyelinating leukodystrophy known as Pol III-related leukosystrophy [105]. Interestingly, also differentially methylated are *SOX8* and *NFIA*, which is directly regulated by another SOX family member (*SOX9*). *NFIA* and *SOX9* have been described as critical transcription factors in the onset of gliogenesis [106, 107] whereas *SOX8* has been described as a multiple sclerosis risk gene [108], with a critical role in oligodendrocyte terminal differentiation and myelin maintenance [109], further hinting at epigenetics potentially contributing to impaired oligodendroglial signatures and function, which are especially relevant in FTLD [10, 110, 111]. On the other hand, differentially methylated genes in CER include several transcription factors implicated in early developmental stages and critical processes for brain patterning and cerebellum formation, such as *BARHL1* [112], *OTX1* and *OTX2* [113]. Interestingly, aberrant expression of another subset of homeobox genes (Hox genes) has already been described in FTLD, specifically in CER from TDP-C9, where (re)activation of such genes in the adult brain was proposed as a compensatory mechanism of reduced *C9orf72* expression [114]. Hox genes were not differentially methylated in the sporadic groups that we investigated however, the fact that we do identify several other homeobox-containing genes in these patients suggests that dysregulation in adult brain of otherwise developmental genes might be a common feature of early stages of FTLD. Additional studies to investigate the underlying mechanisms and the contribution of aberrant methylation patterns are therefore needed.

From our DMR analyses, although we identified several differentially methylated genes, we only detected differential expression of a small fraction of these genes. We attribute this observation to several potential factors: *(i)* some DMRs may have no effect or several layers of regulation exist for the target gene that could compensate for the methylation changes; *(ii)* only some CpG sites drive the effect within the larger DMR and thus additional refinement of the test regions are needed; *(iii)* depending on the DMR location, the methylation changes may alter the expression level and/or ratio of specific gene transcripts; *(iv)* the closest annotated gene is not the target gene; *(v)* methylation changes at the DMR may affect splicing rather than gene expression; *(vi)* dysfunctional epigenetic machinery components that regulate transcription via epigenetic modulation; *(vii)* the methylation change only produces an effect in specific cell types and remains therefore undetected when measuring gene expression in bulk tissue; and/or *(viii)* differences in cell type composition, either between patients and controls or between the two studies (i.e. grey versus white matter proportion during tissue dissection). We acknowledge that the lack of cell type information constitutes a weakness of our study and given the significantly different cell type compositions within brain tissue from different pathological subgroups, we cannot exclude the possibility that a fraction of our results is driven by cell type proportion differences. Single-cell methylation studies have been performed in brain tissue from neurotypic controls [115, 116]; however, the different nature of the profiling assays used in these studies compared to RRBS and the lack of coverage within cell type-defining CpGs prevents us from performing cell-type deconvolution or correction of our data. Undertaking single-cell RRBS studies will be needed in the future, as they will provide an invaluable reference dataset for the field and allow us to account for such proportional differences. Furthermore, undertaking single-nuclei transcriptomic studies in human brain tissue, aimed at profiling pathological subtypes of FTLD, will also be of utmost value to disentangle cell type-specific effects of the methylation changes we identify. Lastly, by using end stage disease tissue, we are also unable to determine causality of our findings. Additional studies employing for example engineered CRISPR/dCas9 systems to modulate methylation levels at specific regions will be needed to elucidate the cause-effect of aberrant methylation patterns. An example of a locus that would greatly benefit from additional in-depth studies is *GFPT2*, one of our top findings in FCX, which we validated using the gold-standard bisulfite sequencing approach. Within *GFPT2*, we identified a hypomethylated DMR that did not correlate with gene expression however, as a key enzyme in the hexosamine biosynthesis pathway (HBP), *GFPT2* is a highly important finding. *GFPT2* is the rate limiting enzyme in the HBP, whose end product uridine diphosphate N-acetyl glucosamine (UDP-GlcNAc) is required for both O-GlcNAcylation and O- and N-linked glycosylation of proteins [117, 118]. O-GlcNAcylation, a post-translation modification, is particularly abundant in the brain, which plays a critical role in regulating diverse neural functions and whose impairment has been associated with aging and several neurodegenerative diseases including AD, Huntington’s, Parkinson’s and ALS [117, 118]. Several neurodegenerative disease proteins have been shown to be O-GlcNAcylated such as tau, α-synuclein, huntingtin, and more importantly, TDP-43 [117, 118]. In the context of ALS, a previous study reported that O-GlcNAcylation of TDP-43 prevents its aggregation and hyperphosphorylation, as well as promoting its RNA splicing activity [119]. Given its critical role in modulating phosphorylation and pathological protein aggregation, the therapeutic value of O-GlcNAcylation and the HBP is being actively explored in the context of several neurodegenerative diseases such as AD [120] and PD [121], and holds great potential in the context of FTLD and TDP-43 proteinopathies. However, additional studies are needed to determine the functional relevance of the methylation changes we observe.

From the genes harboring a DMR that correlated with expression, our most significant finding was *CAMTA1* in the TDP-A group, driven by a hypomethylated gene body DMR in these patients. Within this DMR, we identified four CpG sites that drive this effect, possibly by altering chromatin conformation and/or transcription factor affinity in the locus. *CAMTA1* is a transcription factor highly expressed in brain that plays a multitude of roles in the human nervous system, including regulating cell cycle and differentiation [122], long-term memory formation [77], and development, maturation and survival of cerebellar neurons [78]. Variants in *CAMTA1* have been associated with cerebellar ataxia with variable cognitive and behavioral abnormalities (CECBA) [123–125] as well as reduced survival in ALS patients [126, 127]; however, in our cohort we do not observe a correlation between *CAMTA1* methylation and age at onset, age at death or survival (data not shown). Epigenetic regulation of *CAMTA1* has been studied in various contexts, including cancer, where histone modifications have been shown to modulate *CAMTA1* expression [122], and ischemic stroke, where CpGs in the promoter of *CAMTA1* were found to be hypermethylated in peripheral blood from patients compared to controls, and correlated with gene expression [128]. In the context of neurodegenerative diseases and specifically DNA methylation, CpGs within *CAMTA1* were recently found to be hypomethylated in prefrontal cortex from AD cases compared to controls [129]. Importantly, all these studies indicate that loss or impaired *CAMTA1* expression and/or function leads to detrimental outcomes, and as a TDP-43 target [11, 79, 80], differential methylation of *CAMTA1* is an especially interesting finding in FTLD-TDP patients. Our study is in line with this observation; however, our results also show that the methylation changes we observe are independent of TDP-43 dysfunction, suggesting a double-hit mechanism contributing to *CAMTA1* dysfunction in TDP-A patients. Given that *CAMTA1* methylation in the brain as well as cord blood has been shown to be modulated by prenatal tobacco and alcohol exposure, and associated with cognitive development [130, 131], one can speculate that such events alter *CAMTA1* methylation and levels early in life, increasing disease risk in these individuals. At later stages and due to additional risk factors, once TDP-43 dysfunction is established, *CAMTA1* levels may reach a critically low threshold, likely contributing to the pathophysiology of FTLD-TDP type A. In addition to reduced *CAMTA1*, our results also show a concomitant increase in the expression of an additional gene, *VAMP3*, possibly due to methylation changes affecting the function of enhancer elements in the locus. *VAMP3*, also known as cellubrevin, is part of the SNARE family of proteins and more specifically the vesicle SNARE or v-SNARE group of proteins, which control the fusion of vesicles within the cell [132, 133]. *VAMP3* has been implicated in several cellular processes including autophagy, through its role in autophagosome biogenesis [132, 133] and indirectly in neurodegenerative diseases such as AD, due to being regulated by the AD risk factor PICALM (Phosphatidylinositol binding clathrin-assembly protein) [134]. Previous studies have shown that PICALM modulates autophagy by regulating the endocytosis of SNARE proteins such as VAMP3, and that altered autophagy impairs the clearance of substrates such as tau [134, 135]. Furthermore, important roles have been attributed to VAMP3 in myelination processes [136, 137] and inflammation, where VAMP3 KO was shown to attenuate macrophage infiltration and proinflammatory cytokine production in CFA-induced mouse inflammation models [138]. Finally, VAMP3 is also known to synergistically interact with other VAMP family proteins as well as members of the Rab small GTPase family, which play critical roles in the fusion of autophagosomes with lysosomes for content degradation [133, 139, 140]. This potential link to lysosome biology is of utmost importance, as lysosome dysfunction is a hallmark of FTLD and especially relevant in the context of TDP-A patients, where the DMR was identified [141]. An exciting perspective is the possibility of profiling methylation levels at the *CAMTA1* DMR and/or other loci that could serve as potential biomarkers to predict distinct underlying pathologies of clinical FTLD patients, which is desperately needed. Additional more in-depth studies are, however, needed to investigate whether methylation changes that we detect in brain tissue from FTLD-TDP patients can also be detected in more readily available tissues and/or biofluids (i.e. skin or blood). Finally, our results highlighting that epigenetic machinery components may be dysregulated in FTLD-TDP patients and possibly contribute to a proportion of our findings, add to the exciting and promising potential of using epigenetic therapies to target neurodegenerative diseases, an avenue currently being explored by a growing body of research and clinical trials [142]. Future studies will be critical to investigate in depth the (dys)function of these and other epigenetic machinery components and their role in FTLD-TDP and its pathological subtypes, to fully explore and capitalize on the immense potential of this understudied field.

## Conclusions

By performing this large DNA methylation study, we added important insights to our understanding of FTLD-TDP pathophysiology and the contribution of epigenetic variation to the disease. Overall, we identified a large proportion of CpGs and DMRs that are differentially methylated in FTLD-TDP brains, of which the majority was specific to an FTLD-TDP pathological subtype and brain region. We further implicated DNA methylation in the dysregulation of important disease-relevant processes such as protein phosphorylation and DNA damage repair in TDP-A, cholesterol biosynthesis in TDP-B; and protein localization and transcription regulation in TDP-C. Importantly, our findings corroborate the notion that distinct pathomechanisms underlie each individual FTLD-TDP subtype, which could in fact be considered separate diseases. Additional studies are needed to replicate our results and investigate the functional outcomes of modulating methylation levels at the DMRs we identify. In sum, our findings open exciting new avenues to explore the diagnostic and therapeutic potential of new genes implicated in FTLD-TDP pathogenesis such as *GFPT2* and *CAMTA1*.

## Electronic supplementary material

Below is the link to the electronic supplementary material.

Supplementary Material 1

Supplementary Material 2

## Data Availability

Raw methylation and FCX transcriptomic data supporting the conclusions of this article are available through the dbGaP platform under the project title “RNA transcriptomic and DNA methylation landscape in FTLD-TDP and controls” (accession number phs004075.v1.p1). Raw CER transcriptomic data is partly available through dbGaP (accession number phs003065.v1.p1). Processed transcriptomic data used in this study is included as supplementary data (Supp. Tables 11 and 12). Additional information will be available upon reasonable request.

## Abbreviations

5mC: 5-methylcytosine
ABC: Combined patient group including FTLD-TDP types A, B and C
Amp: Ampicillin
BS: Bisulfite sequencing
BP: Biological process
CAMTA1: Calmodulin binding transcription activator 1
CER: Cerebellum
CGI: CpG island
CpG: Cytosines that precede a guanine nucleotide
C9orf72: Chromosome 9 open reading frame 72
DN: Dystrophic neurite
DMR: Differentially methylated region
FCX: Frontal cortex
FDR: False discovery rate
FTLD: Frontotemporal lobar degeneration
FTLD-TDP: FTLD with TDP-43 pathology
GFPT2: Glutamine-fructose-6-phosphate transaminase 2
GO: Gene ontology
GRN: Progranulin
HBP: Hexosamine biosynthesis pathway
IPTG: Isopropylthio-β-galactoside
KD: Knockdown
NCI: Neuronal cytoplasmic inclusion
MAF: Minor allele frequency
MF: Molecular function
MOI: Multiplicity of infection
ONT: Oxford Nanopore Technologies
PBMC: Peripheral blood mononuclear cell
PCA: Principal component analysis
QC: Quality control
RIN: RNA integrity number
RRBS: Reduced representation bisulfite sequencing
shRNA: Short hairpin RNA
SNP: Single nucleotide polymorphism
TDP: Combined group including all FTLD-TDP patients
TDP-43: TAR DNA-binding protein 43
TDP-A: FTLD-TDP type A
TDP-B: FTLD-TDP type B
TDP-C: FTLD-TDP type C
TDP-GRN: FTLD-TDP patients carrying a *GRN* mutation
TDP-C9: FTLD-TDP patients carrying a repeat expansion in *C9orf72*
TSS: Transcriptional start site
UDP-GlcNAc: Uridine diphosphate N-acetyl glucosamine
UTR: Untranslated region
VAMP3: Vesicle-associated membrane protein 3
X-Gal: 5-bromo-4-chloro-3-indolyl-beta-D-galacto-pyranoside
