# Supplementary material for: Methylome analysis of FTLD patients with TDP-43 pathology identifies epigenetic signatures specific to pathological subtypes
Source: Mol Neurodegener. 2025 Jul 6;20:80. doi: 10.1186/s13024-025-00869-2 (PMC12232778; doi:10.1186/s13024-025-00869-2)
Supplement: Supplementary file 1 — Supplementary Material 1 [file 13024_2025_869_MOESM1_ESM.pdf]

Methylome analysis of FTLD patients with TDP-43 pathology identifies epigenetic signatures specific to pathological subtypes

Cristina T. Vicente[1,2], Tejasvi Niranjan[1,2], Elise Coopman[1,2], Júlia Faura[1,2], Sara Alidadiani[1,2], Claudia Schrauwen[1,2], Billie J. Matchett[3,4], Bavo Heeman[1,2], Marleen Van den Broeck[1,2], Wouter De Coster[1,2], Thuy Nguyen[4], Julie S. Lau[5], Saurabh Baheti[6], Tim de Pooter[7,8], Peter De Rijk[7,8], Mojca Strazisar[7,8], Matt Baker[4], Mariely DeJesus-Hernandez[4], NiCole A. Finch[4,9], Cyril Pottier[1,2,10,11], Marka van Blitterswijk[4], Yan Asmann[12], Melissa E. Murray[4], Leonard Petrucelli[4], Andrew King[13,14], Claire Troakes[13], Safa Al-Sarraj[13,15], Robert A. Rissman[16,17], Annie Hiniker[18], Margaret Flanagan[19], Bret M. Evers[20], Charles L. White III[20], Carlos Cruchaga[21], Rudolph Castellani[22], Jeroen G.J. van Rooij[23], Merel O. Mol[24], Harro Seelaar[23], John C. van Swieten[23], Björn Oskarsson[25], Robert Ross Reichard[26], Aivi T. Nguyen[26], Keith A. Josephs[27], Ronald C. Petersen[27], Nilüfer Ertekin-Taner[4,25], Bradley F. Boeve[27], Neill R. Graff-Radford[25], Sarah Weckhuysen[,2,28,29], Dennis W. Dickson[4], Rosa Rademakers[1,2,4*]

*** Corresponding Author**

Rosa Rademakers, Ph.D. (ORCID: 0000-0002-4049-0863)

VIB Center for Molecular Neurology

Universiteitsplein 1, 2610 Wilrijk, Belgium

Phone: +32 3 265 95 95

E-mail: rosa.rademakers@uantwerpen.vib.be

Supplementary figure legends

Supplementary Figure 1. Quality control of RRBS data. Imputed sex scores for each sample, which was estimated by calculating the ratio between normalized coverage over the Y chromosome against the normalized coverage for the X chromosome. Shown are the standardized ratios for each sample against the mean across all samples. Scores above 1 demonstrate a male pattern of chromosomal coverage, and scores below 1 demonstrate a female pattern of chromosomal coverage. Three samples that were wrongly ascertained were removed from further analyses **(A)**. Principal Components Analysis (PCA) of methylation values. For CpG positions with coverage ≥5 in all samples, a percent methylation value was derived for each sample, and used for PCA and the first six principal components were derived. FCX and CER samples are plotted in red and black, respectively. The first PC fully separates the samples by tissue-of-origin. Samples that inappropriately segregate with the wrong tissue or that constitute outlier samples, were removed from further analyses **(B)**.

Supplementary Figure 2. Most differentially methylated CpG-containing genes are unique to a pathological group. Upset plot showing the number of unique and overlapping genes in each pathological group, considering all genes containing at least one differentially methylated CpG, in FCX **(A)** and CER **(B)**.

Supplementary Figure 3. *NFATC1* is differentially methylated and expressed in FCX from FTLD-TDP patients. Schematic representation depicting the *NFATC1* gene and the location of differentially methylated CpGs (triangles). Blue and red triangles represent CpGs identified in FCX and CER, respectively. Dark (pointing up) and light (pointing down) shades represent hyper- and hypomethylated CpGs, respectively. The purple triangle represents the 5’UTR CpG identified in both FCX and CER. The grey triangle represents the previously identified CpG (cg17938607

[29]). Blue boxes represent exons, lines represent introns, and light blue boxes represent UTRs **(A)**. *NFATC1* expression levels in FCX from controls and FTLD-TDP, with comparison Pvalue using a Mann-Whitney test **(B)**. Pearson correlation between *NFATC1* expression and methylation levels at the 5'-UTR CpG, in FCX from all FTLD-TDP **(C)**.

Supplementary Figure 4. Bisulfite sequencing validates RRBS finding at the *GFPT2* DMR. Upset plot showing the number of unique and overlapping genes in each pathological group, considering all genes containing at least one DMR, in FCX **(A)** and CER **(B)**. Locus view of the *GFPT2* gene in TDP-C patients, depicting the location of the hypomethylated DMR within the gene (black bar inside box), and a zoomed view of the DMR showing all CpGs in the region (blue dots at the top, plotted as percent difference ± SE in average methylation from TDP-C versus controls). Transparency of dots and bars represent the number of samples profiled, with fewer samples (longer error bars) resulting in greater transparency, while more samples (shorter error bars) results in more opacity **(C)**. Results from bisulfite sequencing in control (N=4) and TDP-C (N=4) samples, targeting the *GPFT2* DMR. Dots represent individual CpGs measured (empty= unmethylated; filled= methylated) and rows represent individual colonies profiled. Colored circles in the panel on the right depict methylation levels measured by RRBS (purple, left column) and bisulfite sequencing (BS; green, right column) **(D)**.

Supplementary Figure 5. Epigenetic machinery components are differentially expressed in FTLD-TDP patients. Expression levels of *DNMT1*, *DNMT3A*, *DNMT3B*, *TET1, TET2* and *TET3* in FCX (blue gene names) and CER (red gene names) from all FTLD-TDP patients and controls. Pvalue from each comparison is shown, with ns= not significant **(A)**. Expression levels of the same genes in each pathological group compared to controls, in FCX (blue gene names) and CER (red

gene names). Pairwise comparisons between each TDP subgroup and controls, with Bonferroni correction, are represented by *P<0.05, **P<0.01 and ***P<0.001 **(B)**.

Supplementary Figure 6. Detailed view of relevant clusters of related GO terms. Detailed view of all GO terms in FCX-specific clusters 2 and 37, and side-by-side view of FCX (left, blue) and CER (right, red) from clusters 3 and 13. Identified GO terms are shown in circles, with color representing TDP subgroup (red=TDP-A, blue=TDP-B, green=TDP-C and grey=ABC), and circle sizes representing Pvalue. Arrows represent relationships between related GO terms.

Supplementary Figure 7. Differential expression is observed beyond the pathological group where the DMR was identified. Expression levels in each pathological group compared to controls, for five genes in FCX (*CAMTA1*, *NDUFA10*, *PDZD4*, *SPAG7*, and *WBP2NL*; blue gene names) and four genes in CER (*ATP2B3*, *BBS9*, *OTX2* and *PLD5*; red gene names). Pairwise comparisons between each TDP subgroup and controls, with Bonferroni correction, are represented by *P<0.05, **P<0.01 and ***P<0.001.

Supplementary Figure 8. Methyl-CpG binding proteins are differentially expressed in FTLD-TDP patients. Expression levels of *MBD1*, *MBD2*, *MBD3* and *MECP2* in FCX (top row, blue gene names) and CER (bottom row red gene names) from all FTLD-TDP patients and controls. Pvalue from each comparison is shown, with ns= not significant **(A)**. Expression levels of the same genes in each pathological group compared to controls, in FCX (top row, blue gene names) and CER (bottom row, red gene names). Pairwise comparisons between each TDP subgroup and controls, with Bonferroni correction, are represented by *P<0.05 **(B)**.

Supplementary Figure 9. *CAMTA1* harbors a hypomethylated DMR in TDP-A and is a TDP-43 target. Scheme of the *CAMTA1* gene depicting the location of the hypomethylated DMR (red

box) and a locus view of the *CAMTA1* intron containing the DMR (black bar inside box) in TDP-A patients **(A)**. Zoomed view of the DMR showing all CpGs in the region, profiled by RRBS (blue dots at the top, logFC±SD in TDP-A versus controls) **(B)**. Methylation levels at the *CAMTA1* DMR measured by ONT long-read sequencing in the full cohort (combined validation and replication) of controls (N=50; dark shade box) and TDP-A (N=105; light shade box) **(C)**. Pearson correlation between *TARDBP* and *CAMTA1* expression levels in *TARDBP* knockdown iPSC-derived neurons **(D)**.

Supplementary Figure 10. The *CAMTA1* DMR locus is rich in regulatory elements. Overview of UCSC genome browser data including the *CAMTA1* DMR locus depicting all genes within 1MB of the DMR; enhancer elements from the GeneHancer catalog [84] (light green boxes) or a publicly available dataset [85] (light orange boxes) within the intron that contains the DMR; and a zoomed view of the DMR which overlaps an enhancer element from the GeneHancer catalog (light green box), open chromatin shown as DNAseI clusters (blue boxes; darker shades represent stronger open chromatin signals) and transcription factor binding sides measured by ChIP-seq (grey and black boxes; darker shades represent stronger p-values of the match to that position).

Supplementary Figure 11. *VAMP3* is differentially expressed in FCX from TDP-A and is not a TDP-43 target. Expression levels of *PARK7* in FCX from TDP-A and controls **(A)**. Expression levels of *VAMP3* in FCX from each pathological group compared to controls. Pairwise comparisons between each TDP subgroup and controls, with Bonferroni correction, are represented by *P<0.05, **P<0.01 and ***P<0.001. **(B)**. Pearson correlation between *TARDBP* and *VAMP3* expression levels in *TARDBP* knockdown iPSC-derived neurons **(C)**. ns= not significant.

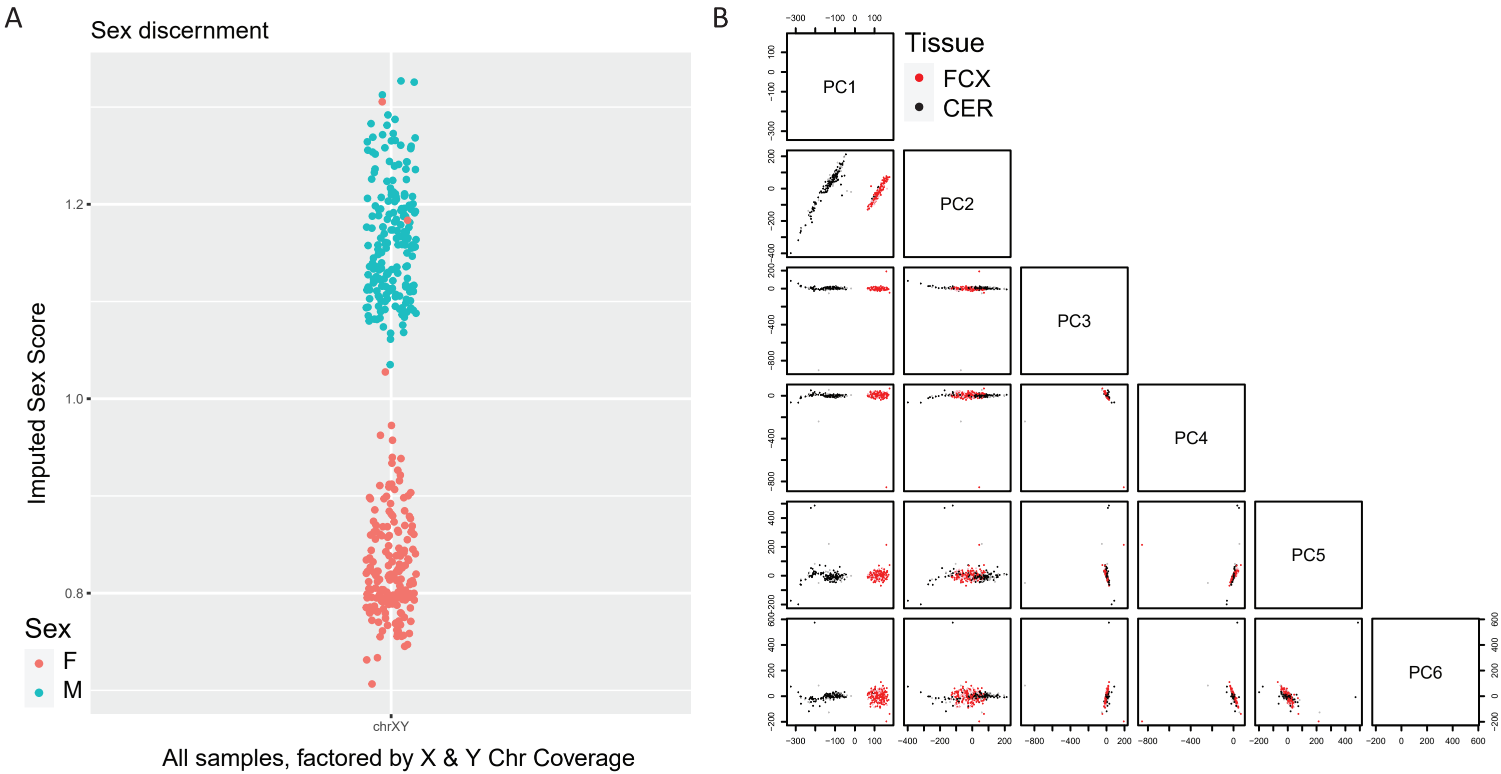

Supplementary Figure 1. Quality control of RRBS data.

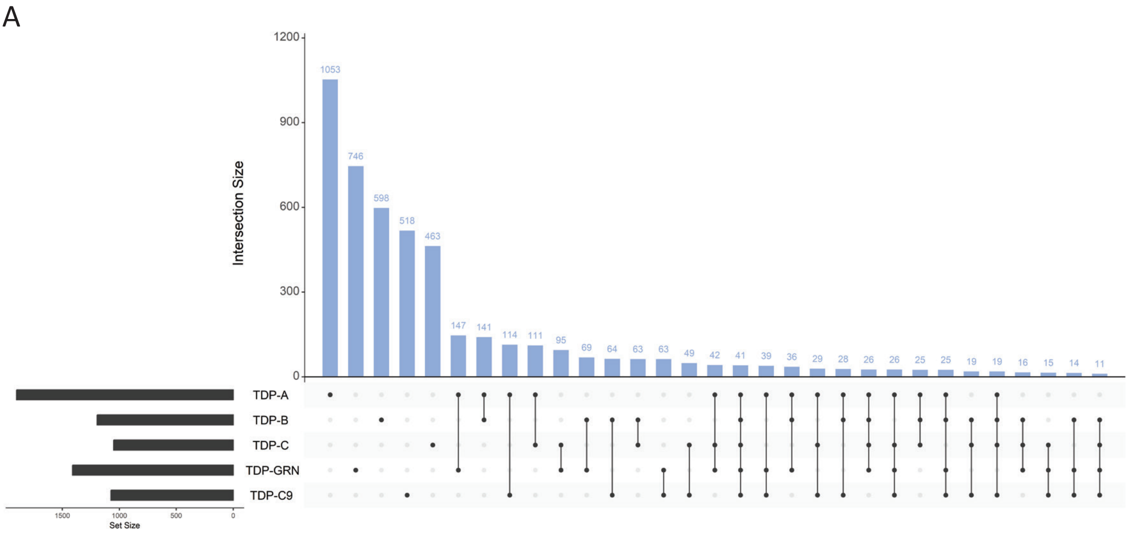

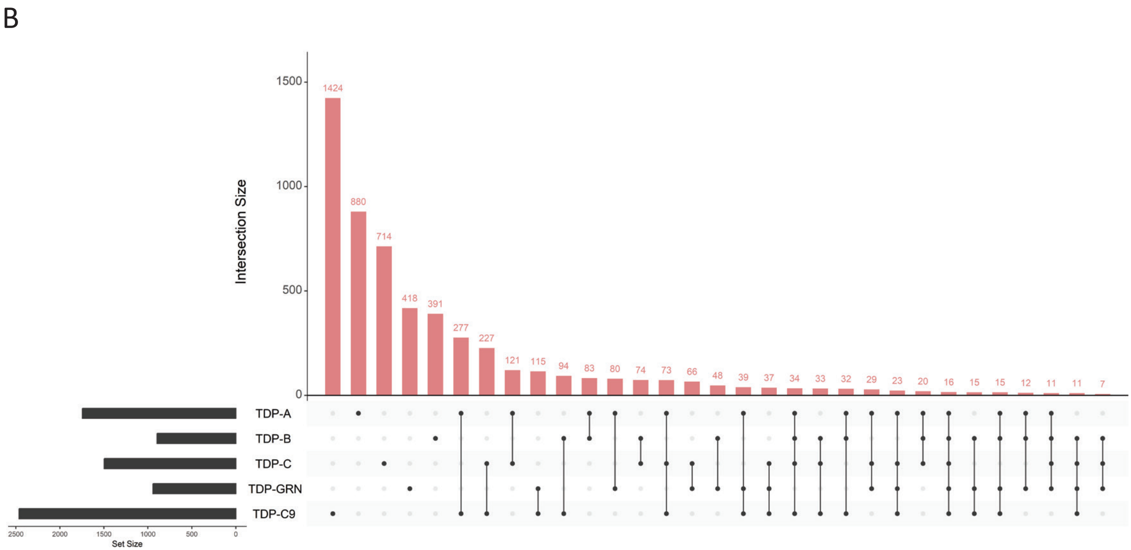

Supplementary Figure 2. Most differentially methylated CpG-containing genes are unique to a pathological group.

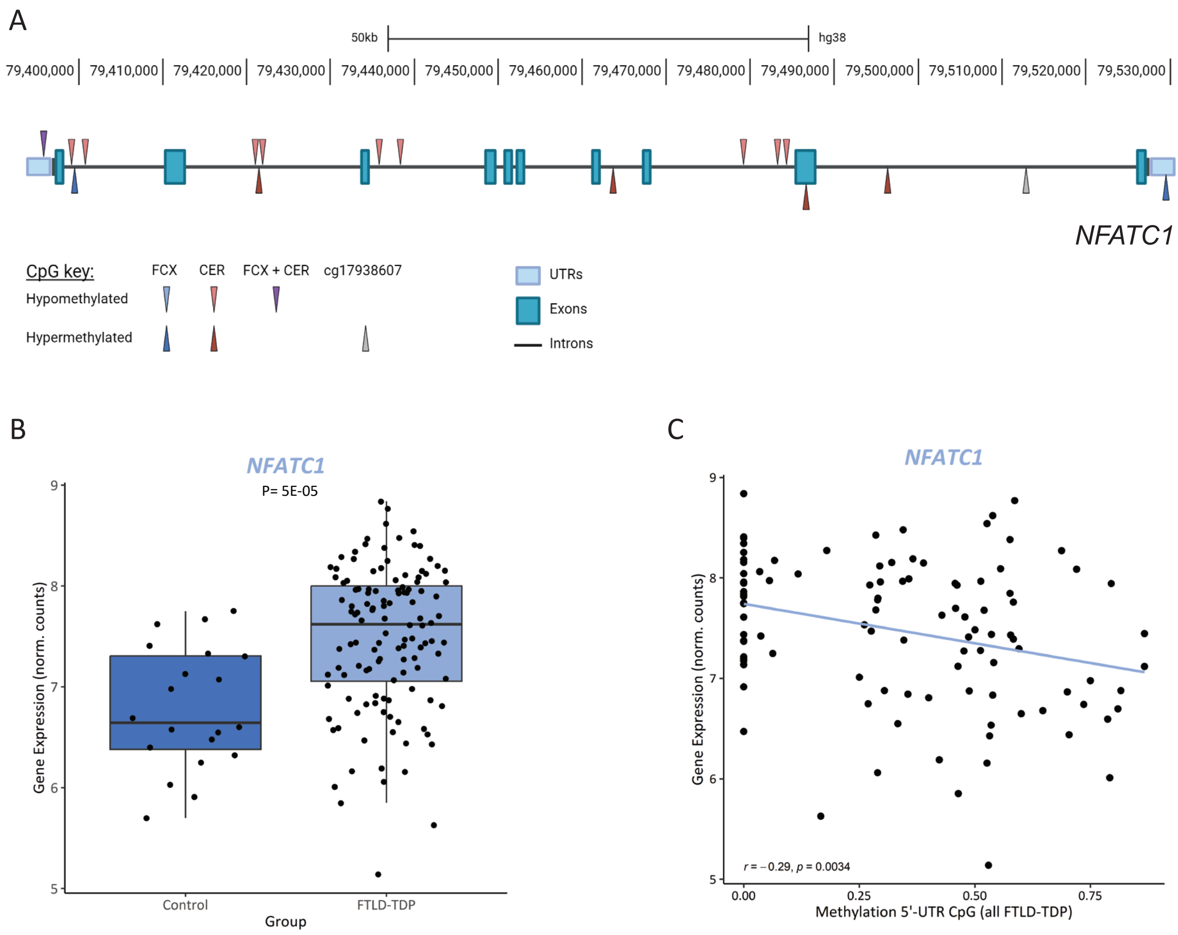

Supplementary Figure 3. ***NFATC1*** **is differentially methylated and expressed in FCX from FTLD-TDP patients.**

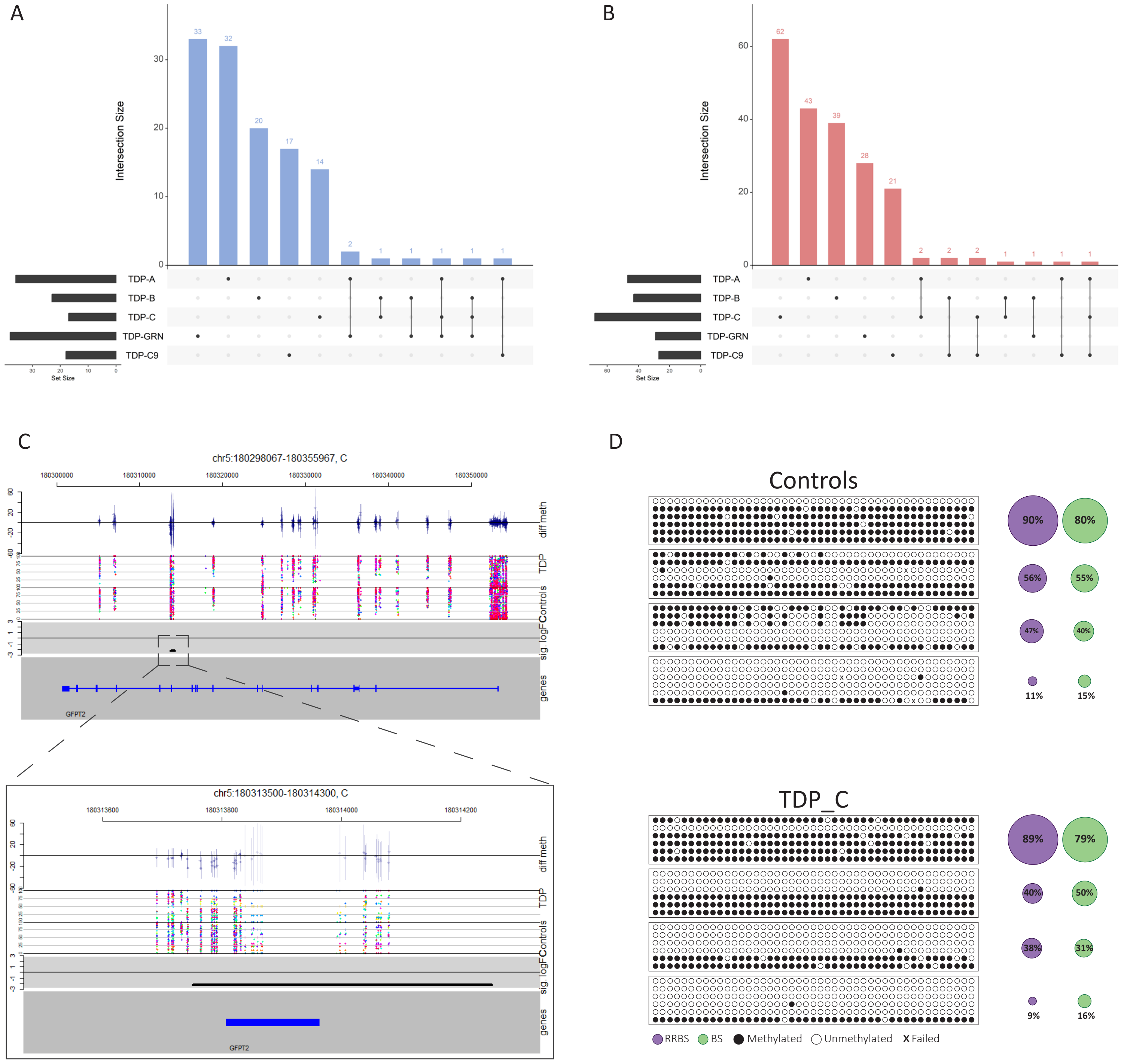

Supplementary Figure 4. Bisulfite sequencing validates RRBS finding at the *GFPT2* DMR.

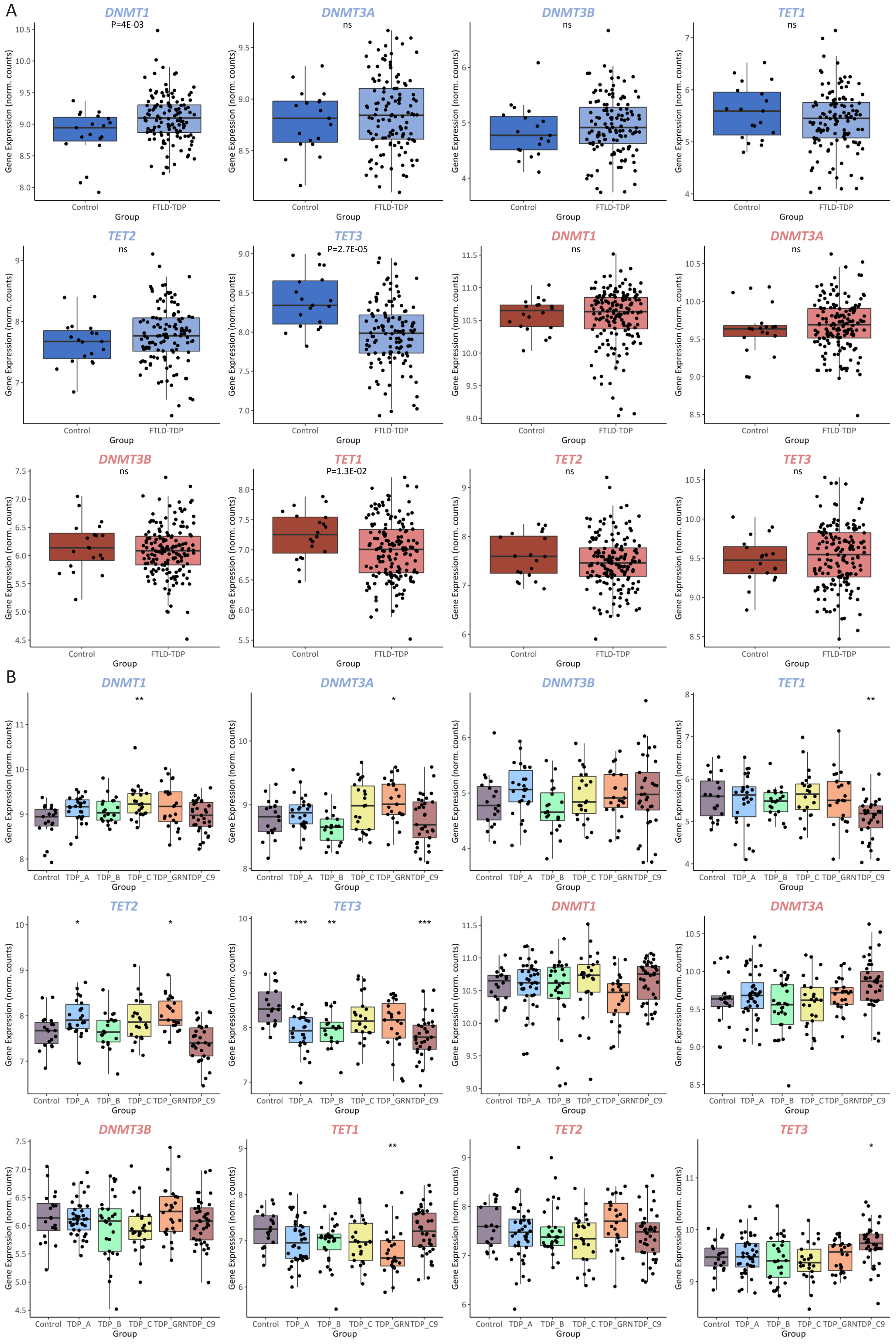

Supplementary Figure 5. Epigenetic machinery components are differentially expressed in FTLD-TDP patients.

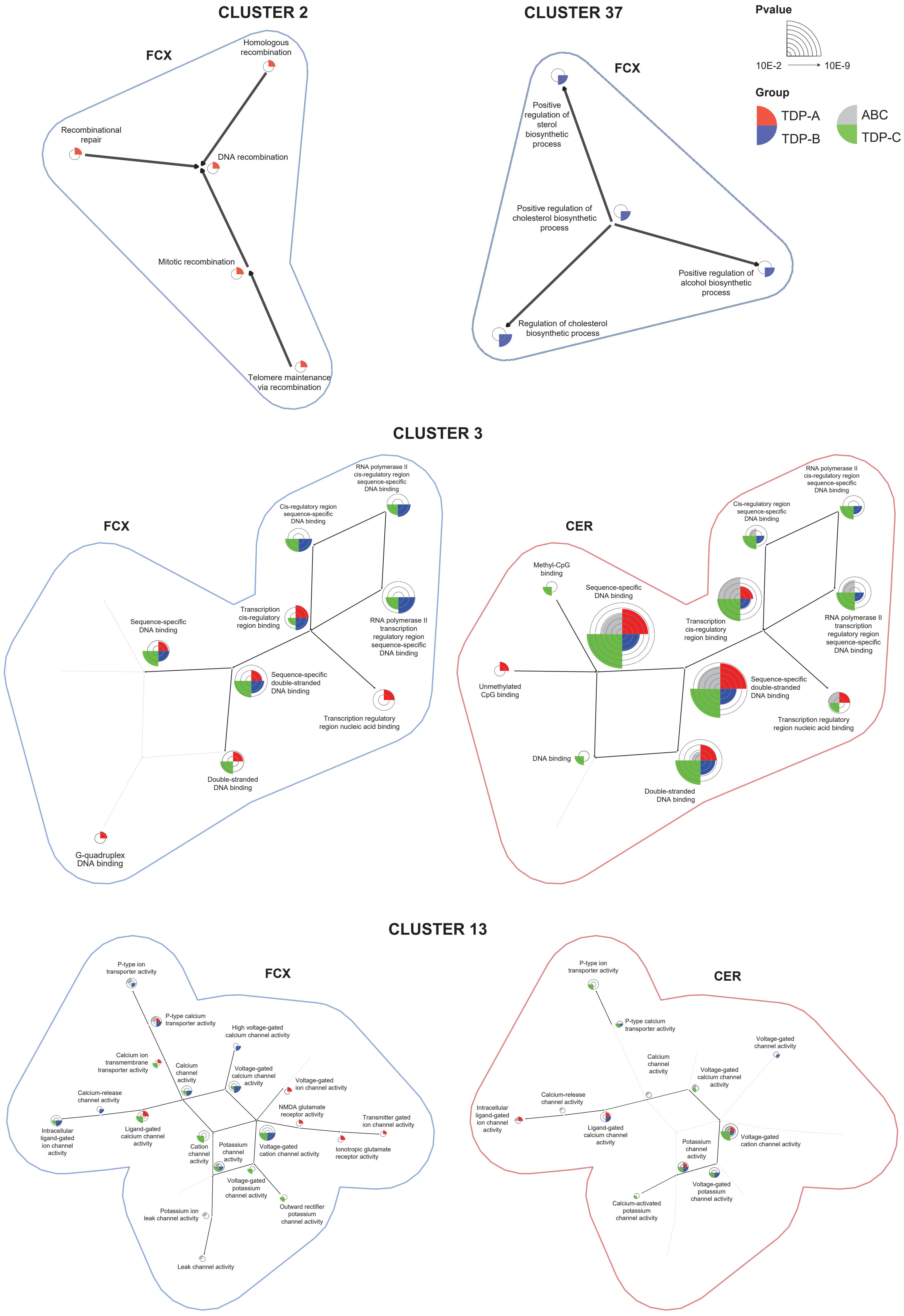

Supplementary Figure 6. Detailed view of relevant clusters of related GO terms.

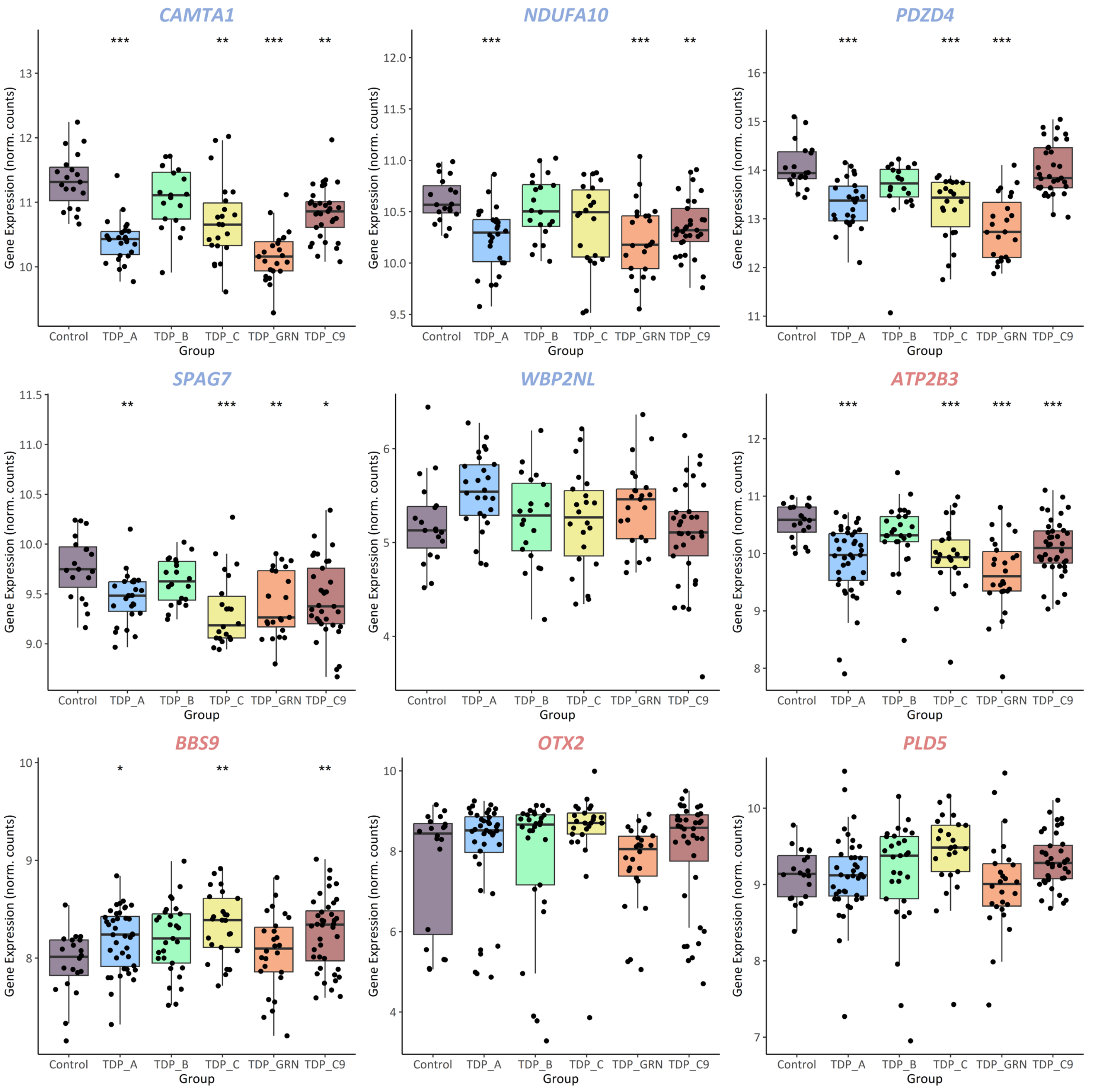

Supplementary Figure 7. Differential expression is observed beyond the pathological group where the DMR was identified.

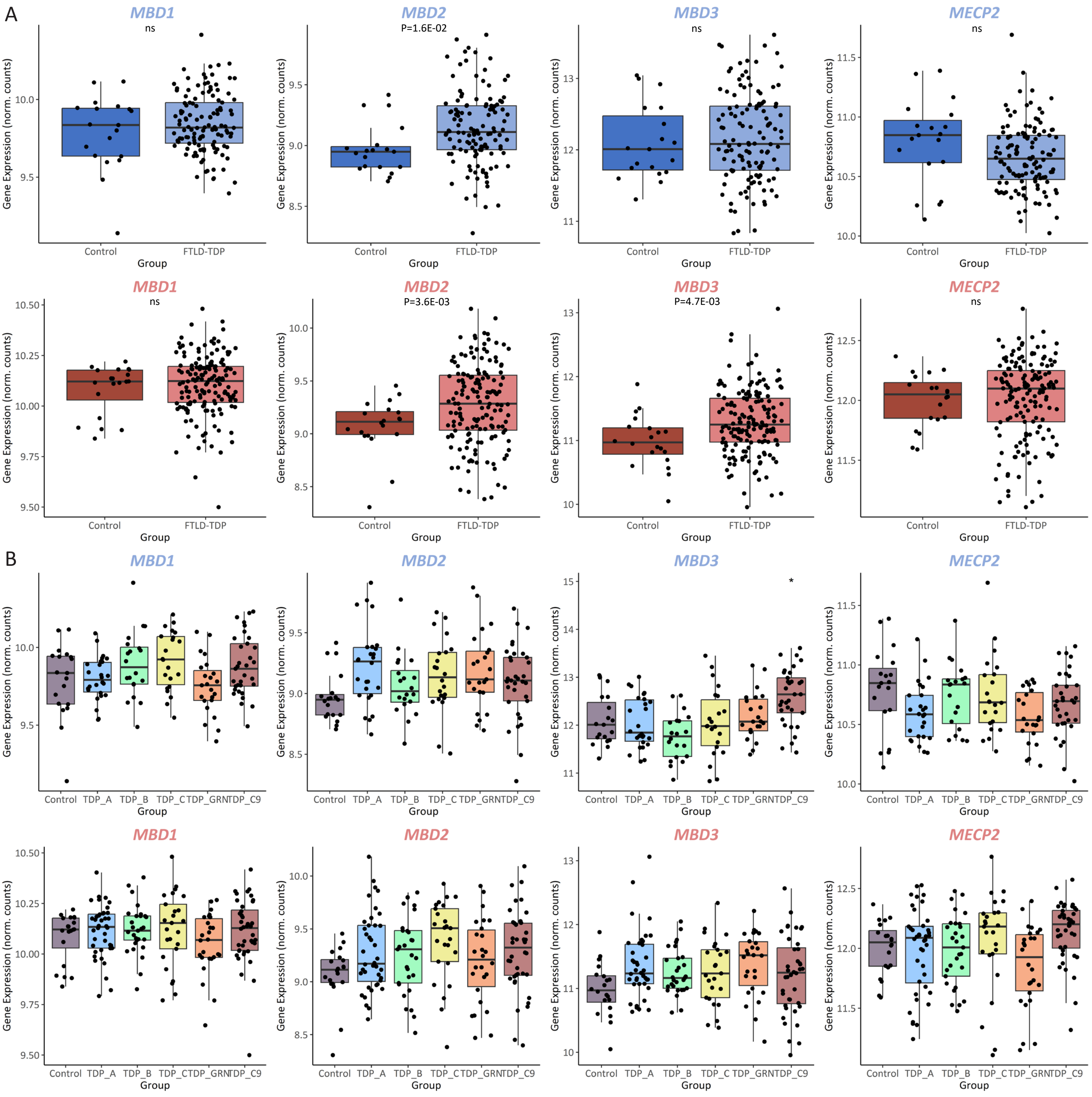

Supplementary Figure 8. Methyl-CpG binding proteins are differentially expressed in FTLD-TDP patients.

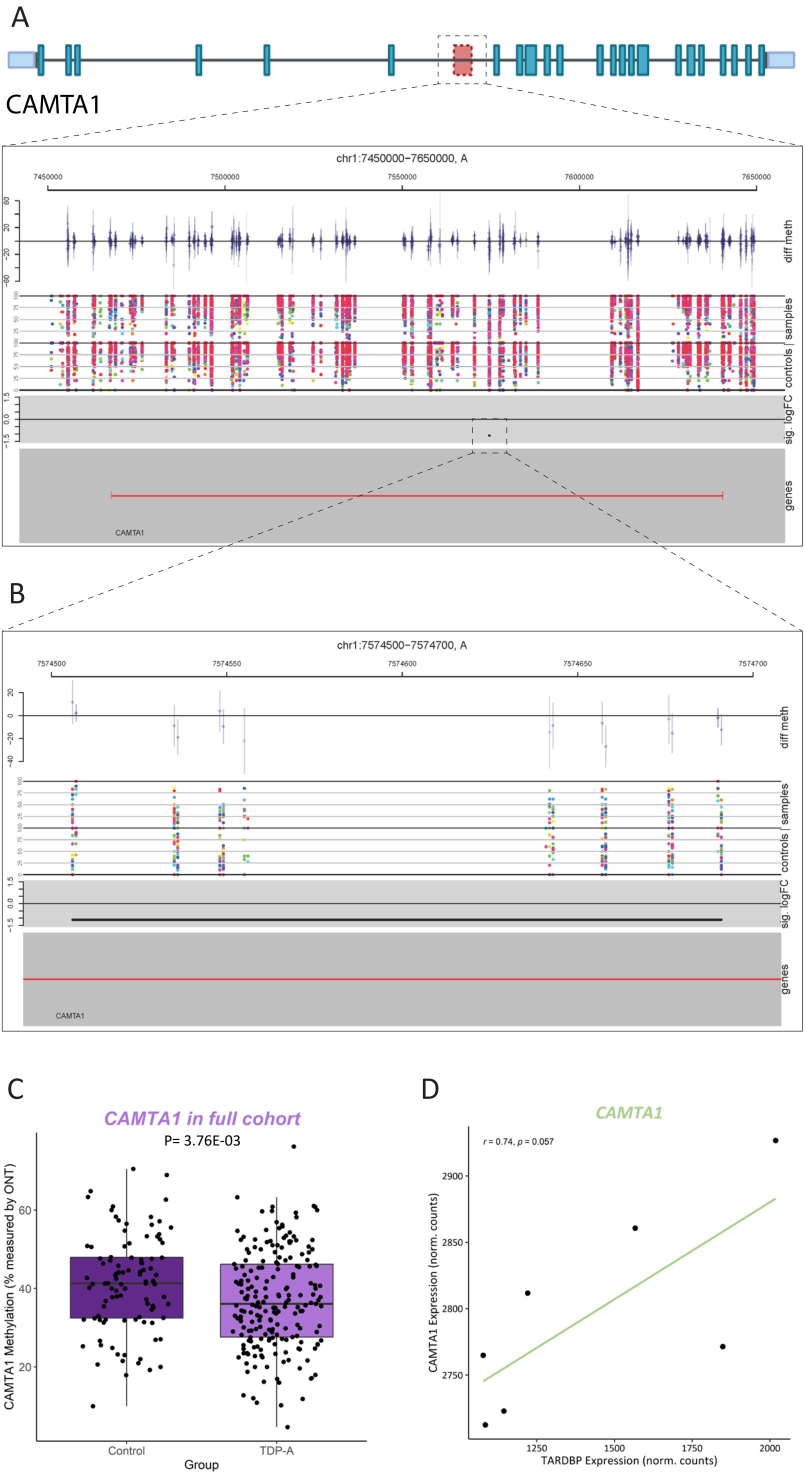

Supplementary Figure 9. CAMTA1 harbors a hypomethylated DMR in TDP-A and is a TDP-43 target.

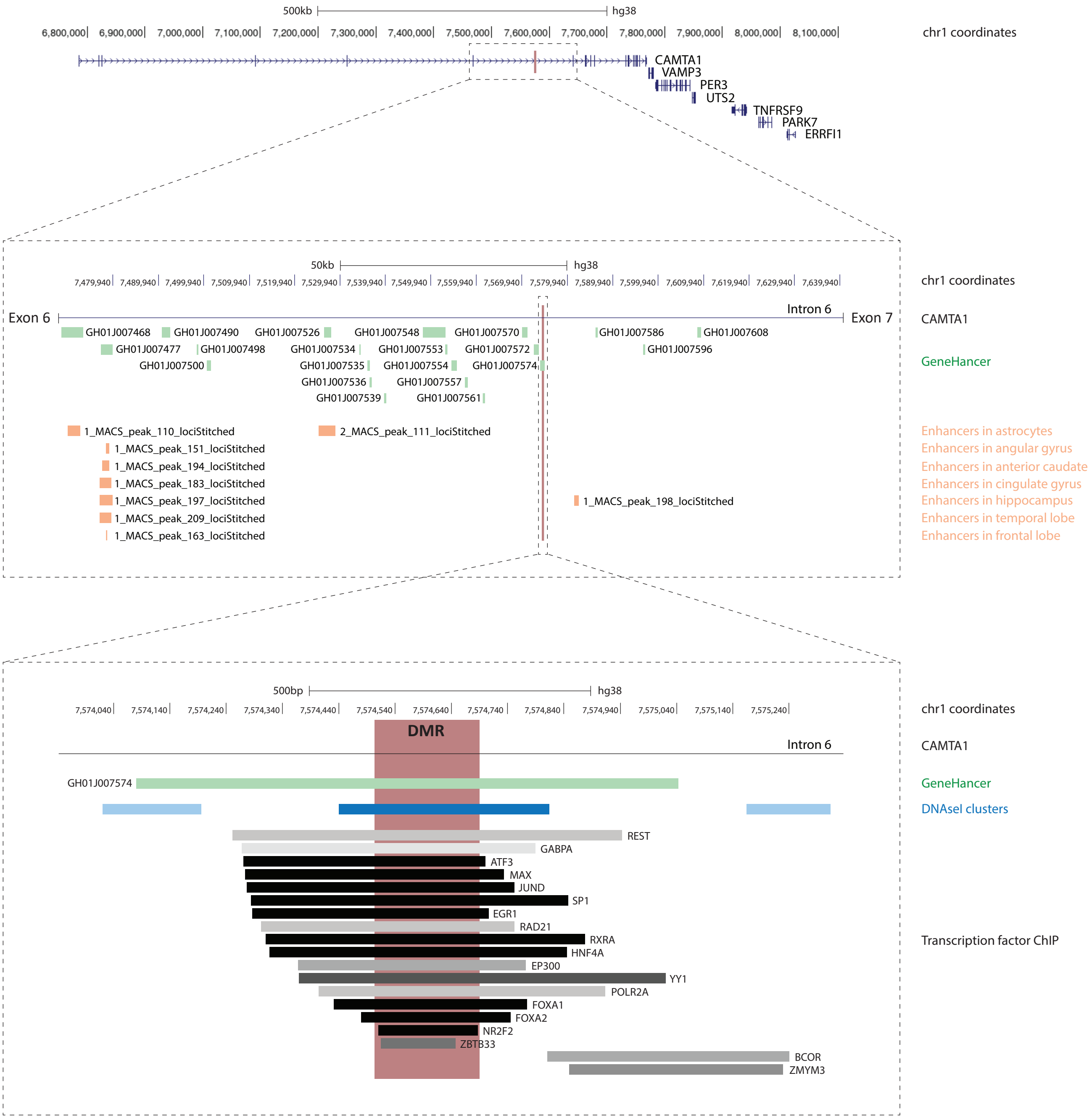

Supplementary Figure 10. The *CAMTA1* DMR locus is rich in regulatory elements.

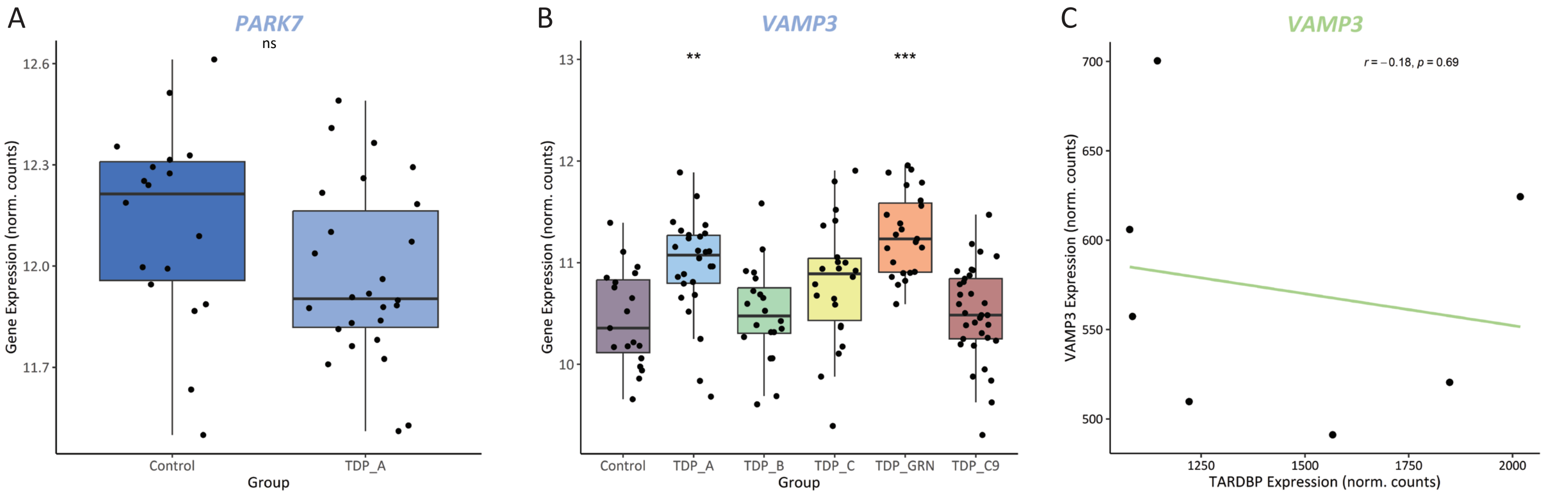

Supplementary Figure 11. ***VAMP3*** **is differentially expressed in FCX from TDP-A and is not a TDP-43 target.**
